# Targeting cuproptosis by zinc pyrithione in triple-negative breast cancer

**DOI:** 10.1016/j.isci.2023.108218

**Published:** 2023-10-16

**Authors:** Xu Yang, Li Deng, Xianhong Diao, Siyuan Yang, Li Zou, Qin Yang, Jian Li, Jianyun Nie, Lina Zhao, Baowei Jiao

**Affiliations:** 1 National Key Laboratory of Genetic Evolution & Animal Models, Kunming Institute of Zoology, Chinese Academy of Sciences, Kunming, Yunnan 650201, China; 2 Kunming College of Life Science, University of Chinese Academy of Sciences, Kunming 650201, China; 3 Department of Breast Cancer, Third Affiliated Hospital, Kunming Medical University, 519 Kunzhou Road, Kunming, Yunnan 650118, China; 4 Institutional Center for Shared Technologies and Facilities, Kunming Institute of Zoology, Chinese Academy of Sciences, Kunming, Yunnan 650201, China; 5 KIZ-CUHK Joint Laboratory of Bioresources and Molecular Research in Common Diseases, Kunming Institute of Zoology, Chinese Academy of Sciences, Kunming, Yunnan 650203, China

**Keywords:** Molecular biology, Cell biology, Cancer

## Abstract

Triple-negative breast cancer (TNBC) poses a considerable challenge due to its aggressive nature. Notably, metal ion-induced cell death, such as ferroptosis, has garnered significant attention and demonstrated potential implications for cancer. Recently, cuproptosis, a potent cell death pathway reliant on copper, has been identified. However, whether cuproptosis can be targeted for cancer treatment remains uncertain. Here, we screened the US Food and Drug Administration (FDA)-approved drug library and identified zinc pyrithione (ZnPT) as a compound that significantly inhibited TNBC progression. RNA sequencing revealed that ZnPT disrupted copper homeostasis. Furthermore, ZnPT facilitated the oligomerization of dihydrolipoamide S-acetyltransferase, a landmark molecule of cuproptosis. Clinically, high expression levels of cuproptosis-related proteins were significantly correlated with poor prognosis in TNBC patients. Collectively, these findings indicate that ZnPT can induce cell death by targeting and disrupting copper homeostasis, providing a potential experimental foundation for exploring cuproptosis as a target in drug discovery for TNBC patients.

## Highlights

• ZnPT induces cuproptosis by disrupting copper homeostasis and DLAT oligomerization
• ZnPT induces cuproptosis *in vivo* and potentially contributes to chemosensitivity of TNBC
• ZnPT inhibits progression and stemness of TNBC

## Graphical abstract

**Figure:**
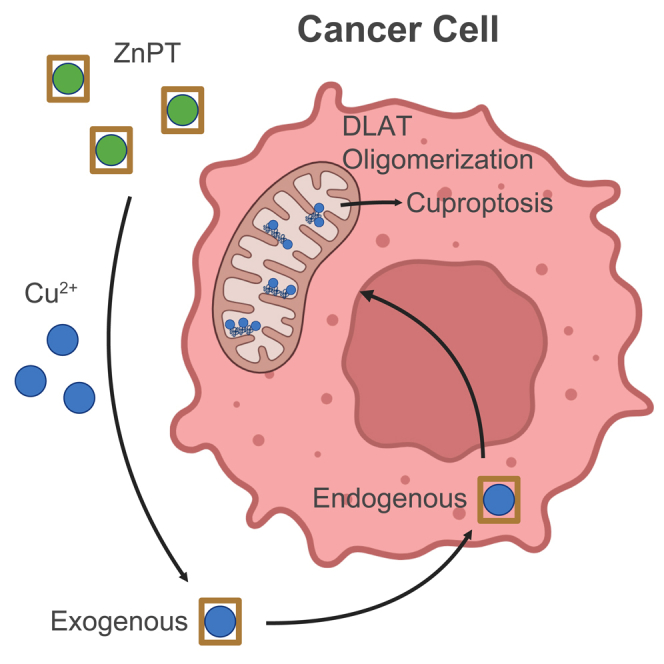

## Introduction

Among tumors in females, breast cancer has the highest incidence and second-highest lethality rate.^1^ It accounts for 10%–20% of total breast cancer cases and exhibits a higher prevalence in younger women.^2^ Compared to other breast cancer subtypes, triple-negative breast cancer (TNBC) is associated with high recurrence rates, high incidence of distant metastases, and poor overall survival (OS).^3^ Despite advancements in cancer therapy, effective TNBC treatment remains a formidable challenge due to its high recurrence and metastatic nature. Thus, there remains a pressing need to explore and develop promising and sensitive therapeutic regimens for TNBC.

Cell death has long been observed in cancers and linked to cancer therapy, with radiation and chemotherapy designed to induce malignant cell death. Understanding the mechanisms of cell death has facilitated the development of drugs that directly activate the cell death machinery and led to improved outcomes in cancer patients. Distinct from accidental cell death (ACD), regulated cell death (RCD), also known as programmed cell death (PCD),^4^ refers to the autonomous and orderly death of cells orchestrated by various biomacromolecules.^4^ Notable types of RCD include apoptosis, necroptosis, pyroptosis, and ferroptosis. Pharmacologically targeting RCD with small molecule compounds has emerged as a promising therapeutic approach,^5^ with rapid advancements in TNBC therapy.^6^ Recently, a novel form of RCD induced by copper ions was discovered, demonstrating cytotoxicity when intracellular copper ion levels surpass the threshold required for maintaining homeostatic mechanisms.^7^ Notably, researchers observed that copper ions directly bind to lipoylated components of the tricarboxylic acid (TCA) cycle, leading to the oligomerization of lipoylated proteins and loss of iron-sulfur cluster proteins, thus triggering proteotoxic stress and ultimately cell death.^7^ This cuproptosis pathway is marked by an abundance of lipoylated proteins, such as dihydrolipoamide branched chain transacylase E2 (DBT), glycine cleavage system protein H (GCSH), dihydrolipoamide S-succinyltransferase (DLST), and dihydrolipoamide S-acetyltransferase (DLAT).^8^ The lipoylation of these proteins is governed by various enzymes, including lipolytransferase 1 (LIPT1), lipoyl synthase (LIAS), and dihydrolipoamide dehydrogenase (DLD),^9^ as well as ferredoxin 1 (FDX1), which functions as a primary upstream regulator of this lipoylation process.^7^ The presence of lipoylation-related enzymes and lipoylated proteins is highly correlated with multiple human tumors. Consequently, copper ionophores may offer therapeutic potential for various cancers with these metabolic traits, although specific compounds for cuproptosis are yet to be identified.

Zinc pyrithione (ZnPT) was originally developed as an effective control measure against the scalp fungus *Malassezia*, which causes dandruff.^10^^,^^11^^,^^12^^,^^13^ Currently, ZnPT holds US Food and Drug Administration (FDA) approval as a topical antimicrobial agent for the treatment of psoriasis and ultraviolet (UV) radiation-induced epidermal hyperplasia.^14^ ZnPT exhibits zinc ionophore properties, which can lead to elevated intracellular zinc levels in yeast,^15^^,^^16^ resulting in mismetallation and cellular stress.^17^ ZnPT can also act as a copper ionophore, inducing intracellular copper influx and iron-sulfur protein inactivation,^18^ thereby inhibiting fungal growth.^19^ Furthermore, ZnPT exposure can lead to mitochondrial dysfunction,^20^ adenosine triphosphate (ATP) depletion, and membrane depolarization.^21^^,^^22^^,^^23^^,^^24^ Thus, due to its broad antimicrobial activity and long history of safe and effective use, recent studies have suggested the therapeutic potential of ZnPT for cancer intervention.^25^^,^^26^^,^^27^^,^^28^^,^^29^

In this study, we observed a significant enrichment of metal ion transport and binding in TNBC patients, alongside elevated expression of cuproptosis-related genes. Among the various antineoplastic agents tested, ZnPT emerged as one of the most promising candidates by facilitating the oligomerization of DLAT, a hallmark feature of cuproptosis. Collectively, our results demonstrated that TNBC patients exhibited heightened sensitivity to cuproptosis and ZnPT. Notably, ZnPT was capable of inducing cuproptosis and significantly inhibiting breast cancer progression.

## Results

### Identification of ZnPT as a potential inhibitor of TNBC cells based on library screening

**Figure 1:**
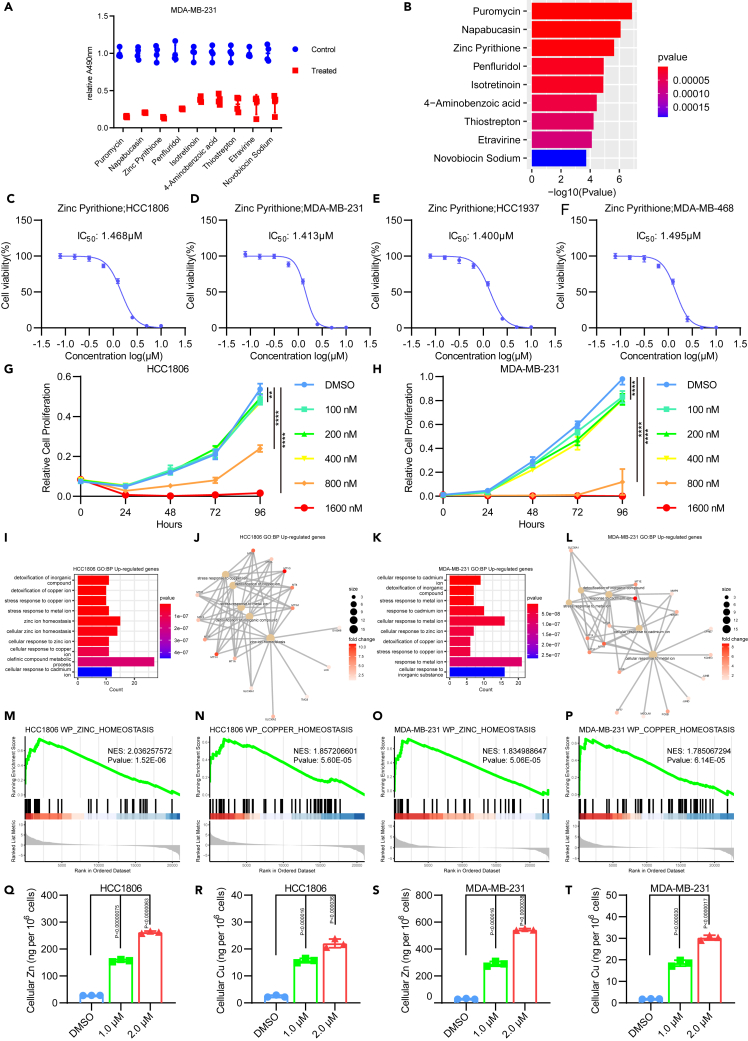
ZnPT dysregulates copper homeostasis and inhibits cell viability and proliferation in TNBC cells (A) Top 10 drugs after using cell viability assay in MDA-MB-231 cells. (B) p value of top 10 drugs ranked by significance. (C–F) TNBC cell viability after ZnPT treatment. (G and H) TNBC cell proliferation after ZnPT treatment. (I–L) GO enrichment analysis of upregulated genes in ZnPT-treated TNBC cells. BP: Biological Process. (M–P) GSEA enrichment analysis of differentially expressed genes in ZnPT-treated TNBC cells. (Q and S) ICP-MS of intracellular zinc in TNBC under ZnPT treatment. (R and T) ICP-MS of intracellular copper in TNBC under ZnPT treatment. Data were presented as means ± SD. ∗, p < 0.05; ∗∗, p < 0.01; ∗∗∗, p < 0.001; ∗∗∗∗, p < 0.0001, two-tailed t test.

Traditionally, the development of new drugs is characterized by lengthy processes and substantial investments.^30^ Hence, drug repurposing for TNBC treatment is a promising strategy.^31^ To identify potential therapeutic agents for TNBC, we conducted cell proliferation assays using an FDA-approved drug library containing 638 small molecule compounds (Table S1) against the MDA-MB-231 TNBC cell line (Figures S1A–S1O). The well-recognized inhibitory chemicals for tumor growth include puromycin, validating our screening approach (Figures 1A and 1B). Candidates selected on the basis of their inhibitory effect include napabucasin, ZnPT, Penfluridol, etc. (Figures 1A and 1B). In addition to puromycin, a well-characterized drug, we further validated the remaining three drugs by examining the cell viability. Napabucasin demonstrated a half maximal inhibitory concentration (IC50) of ≤1.2 μM in the four TNBC cell lines (Figures S2A–S2D), while ZnPT showed inhibitory effects at 1.4 μM (Figures 1C–1F). Penfluridol displayed variable inhibitory effects at concentrations ranging from 1.5 μM to 5.0 μM (Figures S2E–S2H). Napabucasin, a drug specifically developed to target STAT3 in cancer, is underpinned by relatively comprehensive phenotypic and mechanistic studies.^32^ In contrast, oncological research on ZnPT is not well established. The robust inhibitory effects of ZnPT at 1.6 μM were further confirmed through cell proliferation assays in MDA-MB-231, HCC1806, MDA-MB-468, and HCC1937 cells (Figures 1G, 1H, and S2I–S2J). Based on these results, we selected ZnPT for further investigation.

To further explore the mechanism underlying the inhibitory effects of ZnPT on TNBC proliferation, RNA sequencing (RNA-seq) was performed in ZnPT-treated MDA-MB-231 and HCC1806 cells (Figures S3A–S3D). In the HCC1806 cells, Gene Ontology (GO)^33^ biological process enrichment analysis revealed the downregulation of chromosome segregation and nuclear division (Figures S3E–S3F), both crucial processes for eukaryotic cell mitosis, which is dependent on spindle organization.^34^ This was further validated by GO cellular component enrichment analysis, showing downregulation of the spindle (Figures S3I–S3J), which is essential for the separation of condensed chromosomes during meiosis, a process highly dependent on interactions between microtubules and chromosomal kinetochores.^34^ GO molecular function enrichment analysis similarly verified the downregulation of microtubule binding (Figures S3M–S3N). The cyclin-dependent kinase (CDK)-RB-E2F axis represents a core transcriptional mechanism essential for cell cycle progression. Axis alterations in this pathway occur in nearly all cancers and result in elevated oncogenic E2F activity leading to uncontrolled proliferation.^35^ Based on gene set enrichment analysis (GSEA) of the RNA-seq data, hallmark_E2F_targets showed significant downregulation in the ZnPT-treated TNBC cell lines (Figures S3Q–S3R). Additionally, we observed significant downregulation in cell migration and the extracellular matrix (ECM) in MDA-MB-231 cells (Figures S3G–S3H, S3K–S3L, and S3O–S3P). Thus, these findings suggest that ZnPT is a promising compound to inhibit TNBC cell proliferation.

### ZnPT induces cuproptosis by disrupting intracellular copper homeostasis and DLAT oligomerization

**Figure 2:**
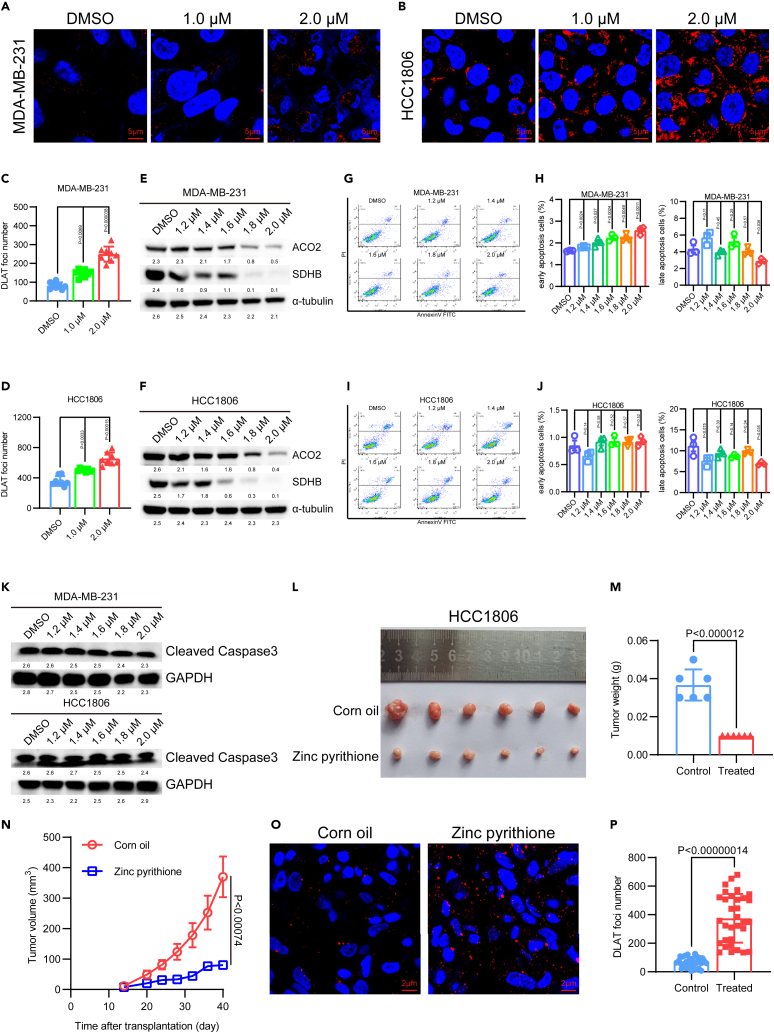
ZnPT promotes oligomerization of DLAT *in vitro* and *in vivo* (A and B) Representative images of immunofluorescence in ZnPT-treated TNBC cells, scale bar 5μm. (C and D) Statistical analysis of DLAT foci number in ZnPT-treated TNBC cells. (E and F) Western blotting of ACO2 and SDHB proteins in ZnPT-treated TNBC cells. (G and I) Representative images of apoptosis cell ratios using flow cytometry. (H and J) Statistical analysis of early and late apoptosis cell ratios in ZnPT-treated TNBC cells. (K) Western blotting of cleaved caspase3 proteins in ZnPT-treated TNBC cells. (L) Image of tumor size at growth endpoint, with each experimental group containing three mice (two tumor blocks in left and right mammary glands of each mouse). (M) Statistical analysis of tumor weight at growth endpoint, with each experimental group containing three mice (two tumor blocks in left and right mammary glands of each mouse). (N) Tumor growth curve of nude mice xenografted with HCC1806 cells with or without ZnPT treatment, with each experimental group containing three mice. (O) Representative immunofluorescence images of DLAT in ZnPT-treated nude mice, scale bar 2μm. (P) Statistical analysis of DLAT foci numbers in ZnPT-treated nude mice. Data were presented as means ± SD. Two-tailed t test.

To explore the mechanism underlying the inhibitory effects of ZnPT on proliferation, we conducted in-depth analysis of the transcriptomic data. Based on GO enrichment analysis, we observed a significant upregulation in detoxification and stress response to copper ions (Figures 1I–1L). The GSEA results validated notable changes in copper homeostasis and response to metal ions (Figures 1M–1P and S2S–S2T). In addition, using inductively coupled plasma mass spectrometry (ICP-MS), we confirmed higher levels of intracellular copper and zinc in the TNBC cell lines following ZnPT treatment (Figures 1Q–1T). Previous research has indicated that dysregulation of copper ions impairs [4Fe-4S] cluster maturation by displacing iron bound to cysteine (Cys) residues with high binding affinity.^36^ Our GO enrichment analysis identified dysregulated expression of genes related to the iron-sulfur protein NADH and NAD(P)H dehydrogenase (Figures S2K–S2L). Copper exposure can induce protein aggregation, leading to protein misfolding.^37^ Similarly, our results showed significant upregulation in misfolded protein binding (Figures S2M–S2N). Additionally, we observed a significant upregulation in autophagosome and phagophore assembly sites (Figures S2O–S2R), consistent with previous studies showing increased autophagy levels in response to copper-carrying molecules.^38^ Recent research reported on a novel form of copper-dependent cell death, known as cuproptosis, which uniquely involves the binding of copper to lipoylation enzymes in the TCA cycle, leading to protein aggregation, proteotoxic stress, and ultimately cell death.^7^ We confirmed these findings through immunofluorescence, observing an increase in DLAT foci with increasing concentrations of ZnPT in TNBC cell lines (Figures 2A–2D and S4A–S4D). We further validated the occurrence of ZnPT-mediated cuproptosis based on analysis of ZnPT-treated HCC1806 and MDA-MB-231 cells using native-polyacrylamide-gel electrophoresis (PAGE) and western blotting assays. Notably, our results showed oligomerization of lipoylated DLAT upon ZnPT treatment in HCC1806 and MDA-MB-231 cells (Figures S4N–S4O). In addition to oligomerization of DLAT, another marker of cuproptosis is the loss of F-S cluster proteins. Consistent with previous findings, treatment with ZnPT significantly downregulated F-S cluster proteins ACO2 and SDHB (Figures 2E, 2F, and S4E–S4F).

Cell death entails complex signaling cascades and well-defined molecular effector mechanisms involving proteins and lipids. Previous studies have shown that ZnPT can induce lysosome-dependent apoptotic cell death.^39^ However, our results revealed that ZnPT-induced cell death did not involve the cleavage or activation of caspase3 activity (Figure 2K and S4K), a hallmark of apoptosis.^40^ Flow cytometry assays further supported these findings, demonstrating that ZnPT induced cell death through an apoptosis-independent pattern, as evidenced by Annexin V/PI staining (Figures 2G–2J and S4G–S4J), a sensitive indicator of apoptosis.^41^ These results provide robust evidence for the role of ZnPT in inducing cuproptosis.

### ZnPT induces cuproptosis *in vivo* and shows potential in chemosensitivity for TNBC patients

To investigate the role of ZnPT in regulating cuproptosis *in vivo*, we conducted experiments in nude mice subcutaneously transplanted with HCC1806 cells. Once tumors reached a size (at least 0.1 × 0.1 cm), intraperitoneal injections of 5 mg/kg ZnPT were administered once every four days. Remarkably, the tumors treated with ZnPT exhibited regression, while the control tumors continued to grow (Figures 2L–2M), with all mice showing a partial response to treatment after 24 days (Figure 2N). Furthermore, immunohistochemical staining for Ki67 confirmed the inhibitory effects of ZnPT treatment on proliferation (Figures S4L–S4M). Notably, DLAT oligomerization was observed upon ZnPT treatment (Figures 2O–2P), validating that the observed cuproptosis was mediated by ZnPT. While ZnPT has clinical approval for topical use, its potential side effects when administered as an intraperitoneal injection in mice remain unclear. However, at the doses applied in our experiments, we observed no marked changes in body weight, immune system, or liver and renal function in the mice (Figures S4P–S4W), suggesting it may be a potentially safe approach for tumor treatment.

**Figure 3:**
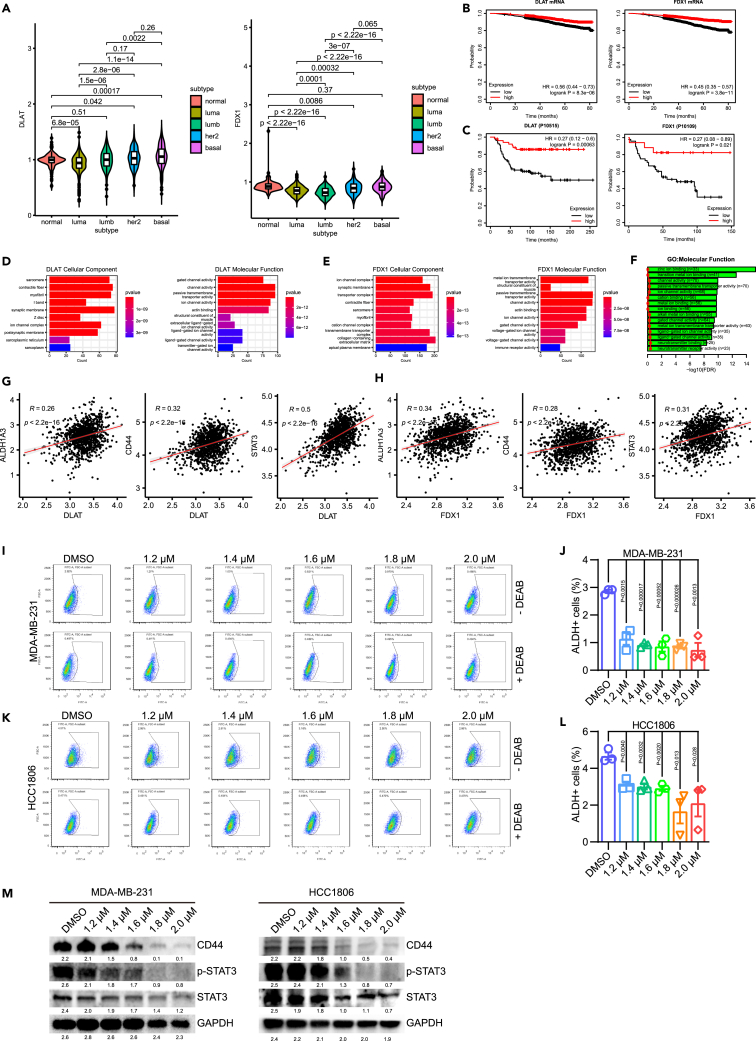
Analysis of cuproptosis-associated genes in breast cancer patients from TCGA database (A) Transcript expression levels of DLAT and FDX1 in normal breast tissue and different breast cancer subtypes from TCGA. (B) Kaplan-Meier survival analysis showing correlation between OS and DLAT and FDX1 mRNA expression. (C) Kaplan-Meier survival analysis showing correlation between OS and DLAT and FDX1 protein expression. (D and E) GO enrichment analysis of upregulated genes in breast cancer patients with high DLAT and FDX1 expression. (F) GO enrichment analysis of differentially expressed genes in breast cancer tissue compared with normal tissues. (G and H) Analysis of TCGA dataset for expression correlation between DLAT and FDX1 and ALDH1A3, CD44, and STAT3. (I and K) Representative images of ALDH+ cell ratios using flow cytometry. (J and L) Statistical analysis of ALDH+ cell ratios in ZnPT-treated TNBC cells. (M) Western blot analysis of CD44, p-STAT3, and STAT3 proteins in ZnPT-treated TNBC cells. Data were presented as means ± SD. Two-tailed t test.

To further assess the clinical benefit of ZnPT in TNBC patients, we analyzed transcriptomic data of TNBC patients obtained from The Cancer Genome Atlas (TCGA) database. Results revealed that proteins within the cuproptosis pathway, specifically those involved in lipoylation such as DLAT and GCSH, were highly expressed in TNBC patients (Figures 3A and S5A). Additionally, upstream regulators of lipoylation, namely FDX1 and DLD, also showed high expression levels in TNBC patients (Figures 3A and S5A). Analysis of OS confirmed that patients with high expression of cuproptosis-related genes at the mRNA and protein levels exhibited poorer outcome (Figures 3B, 3C, and S5B–S5C). Furthermore, GO enrichment analysis indicated that metal ion transmembrane transporter activity and the ion channel complex were significantly upregulated in patients with high expression of cuproptosis-related genes (Figures 3D, 3E, and S5D–S5H). Moreover, zinc ion binding, metal ion binding, and metal ion transmembrane transporter activity were enriched in breast cancer patients (Figure 3G). Overall, this evidence strongly suggests that TNBC patients exhibit chemosensitivity to the cuproptosis pathway under ZnPT treatment.

### ZnPT inhibits TNBC self-renewal and stemness markers

Preclinical studies have shown that disulfiram (DSF) and copper ions in combination can selectively target and kill aldehyde dehydrogenase (ALDH)-positive cancer stem cells (CSCs).^42^^,^^43^^,^^44^^,^^45^ Similarly, our data showed a significant correlation between cuproptosis-related genes and breast cancer stem cell (BCSC) markers (Figures 3F, 3G, and S5I–S5L), suggesting that ZnPT may also target these cells. CSCs are known for their high drug efflux properties, which contribute to their resistance to anticancer drugs.^46^ Additionally, CSCs contain abundant antioxidant molecules, such as glutathione (GSH), which play crucial roles in maintaining homeostasis, stemness, proliferation, and survival.^47^ According to previous studies, mitochondrial GSH can slow copper ion-induced cell death by inhibiting enzymatic lipoylation and oligomerization of DLAT.^48^ These unique properties of CSCs have paved the way for the discovery of small molecule drugs and advancements in targeting these cells.

Previous studies have shown that ALDH, a marker found in many CSCs,^49^ reduces oxidative stress and increases breast cancer resistance to chemotherapeutic agents.^50^ In our study, we observed a remarkable reduction in ALDH expression in the TNBC cell lines upon treatment with 2.0 μM ZnPT (Figures 3H–3K and S5N–S5Q). The transmembrane protein CD44, another major BCSC surface marker, has also been implicated in metastasis, recurrence, and chemoresistance.^51^ CD44 promotes the expression of multidrug resistance genes, and its suppression is associated with enhanced chemosensitivity in cancer cells.^52^ Our analysis confirmed a significant suppression of CD44 protein levels with increasing concentrations of ZnPT (Figures 3M and S5R). In addition to ALDH and CD44 being universally acknowledged as BCSC markers, the STAT3 transcription factor is also widely recognized to govern BCSC behavior.^53^ Interestingly, a significant fraction of STAT3 is localized in the mitochondria,^54^ where cuproptosis occurs. Indeed, we found that the correlation between cuproptosis-related genes and STAT3 was higher than that with ALDH and CD44 (Figures 3F, 3G, and S5M). Overall, STAT3 and phosphorylated STAT3 were downregulated after ZnPT treatment (Figure 3L and S5R).

**Figure 4:**
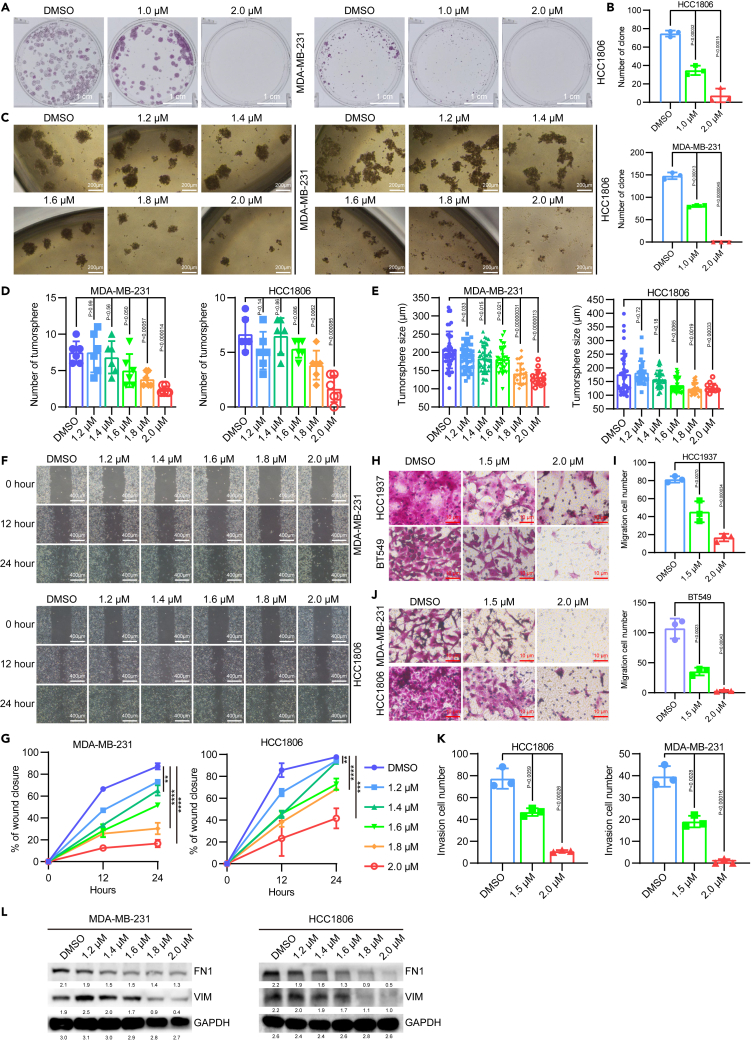
ZnPT represses migration, invasion, and stemness in TNBC cells (A) Representative images of clonal formation assays in ZnPT-treated TNBC cells, scale bar 1cm. (B) Statistical analysis of number of clones in ZnPT-treated TNBC cells. (C) Representative images of tumorspheres in ZnPT-treated TNBC cells, scale bar 200μm. (D) Statistical test of number of tumorspheres in ZnPT-treated TNBC cells. (E) Statistical test of size of tumorspheres in ZnPT-treated TNBC cells. (F) Representative images of wound healing assays in ZnPT-treated TNBC cells, scale bar 400μm. (G) Statistical analysis of healing rate in ZnPT-treated TNBC cells. (H) Representative images of cell migration assays in ZnPT-treated TNBC cells, scale bar 10μm. (I) Statistical analysis of migration cell number in ZnPT-treated TNBC cells. (J) Representative images of cell invasion assays in ZnPT-treated TNBC cells, scale bar 10μm. (K) Statistical analysis of invasion cell number in ZnPT-treated TNBC cells. (L) Western blot analysis of vimentin and fibronectin proteins in ZnPT-treated TNBC cells. FN1: Fibronectin; VIM: Vimentin. Data were presented as means ± SD. ∗, p < 0.05; ∗∗, p < 0.01; ∗∗∗, p < 0.001; ∗∗∗∗, p < 0.0001, two-tailed t test.

To investigate the potential impact of ZnPT on BCSCs, we conducted clonal formation assays, which are commonly used to assess BCSC stemness.^55^^,^^56^ Our results demonstrated that the colony formation ability of the BCSCs was partially inhibited upon 1.0 μM ZnPT treatment and completely repressed upon 2.0 μM ZnPT treatment (Figures 4A, 4B, and S6A–S6B). Tumor spheroids are more resistant to treatment than cells in 2D culture and can recapitulate the drug resistance observed in solid tumors.^57^^,^^58^ The potential anti-CSC agents will be determined by counting the number and the size of formed tumorspheres.^59^ Results showed that ZnPT (2.0 μM) treatment significantly inhibited the number and size of tumorspheres (Figures 4C–4E and S6C–S6E). Thus, these results suggest that ZnPT plays a critical role in inhibiting BCSC stemness.

### ZnPT represses TNBC capability of migration and ECM

Despite significant advancements in cancer diagnosis and treatment, metastasis continues to pose a major challenge in achieving favorable clinical outcomes, given that more than 90% of cancer-related deaths are attributed to metastatic disease.^60^ Primary tumor metastasis is a complex and multistep process involving local migration and invasion of cancer cells into adjacent tissues at the primary site, followed by dissemination into the circulatory system.^61^ Once in the circulation, circulating tumor cells extravasate into distant organs and often remain dormant after colonization. However, in certain cases, dormancy is disrupted, leading to the development of lethal macrometastases.^62^^,^^63^ In the sequential steps in metastasis, dysregulation of ECM homeostasis within solid tumors can have a marked effect.^64^

Based on RNA-seq analysis, our results revealed a significant downregulation in ECM constituents, organization, binding, and cell migration in mesenchymal-like cells MDA-MB-231. In line with these results, we performed a wound healing assay, a classic method for studying directional cell migration *in vitro.*^65^ Results showed that cell migration decreased with increasing concentrations of ZnPT (Figures 4F, 4G, and S6F–S6G). We also conducted a transwell-based assay, commonly used to assess cancer cell migration or invasion capacity.^66^ As anticipated, the ability of the TNBC cells to cross the porous membranes was partially inhibited upon treatment with 1.5 μM ZnPT and completely repressed upon treatment with 2.0 μM ZnPT (Figures 4H–4K and S6H–S6I).

Cell migration relies on the reorganization of microtubules, intermediate filaments, and actin filaments, which form different arrays to support cell propulsion.^67^ Vimentin, a component of intermediate filaments, promotes directional cell migration by coordinating the dynamics of actin filaments and microtubules.^68^^,^^69^ Thus, we measured the expression of vimentin in different ZnPT-treated TNBC lines and found that ZnPT reduced the protein levels of vimentin in a dose-dependent manner (Figures 4L and S6J). Furthermore, fibronectin, a versatile adhesive-like glycoprotein, plays a crucial role in regulating the function and structure of the interstitial ECM as well as cell attachment and migration.^70^ Consequently, we examined the levels of the fibronectin protein and found that they were significantly inhibited with increasing ZnPT concentrations (Figures 4L and S6J). In summary, our findings indicate that ZnPT suppresses wound healing and cell migration in TNBC cell lines, highlighting its potential role in regulating metastasis in TNBC patients.

### TNBC signaling pathways are disrupted by ZnPT treatment

**Figure 5:**
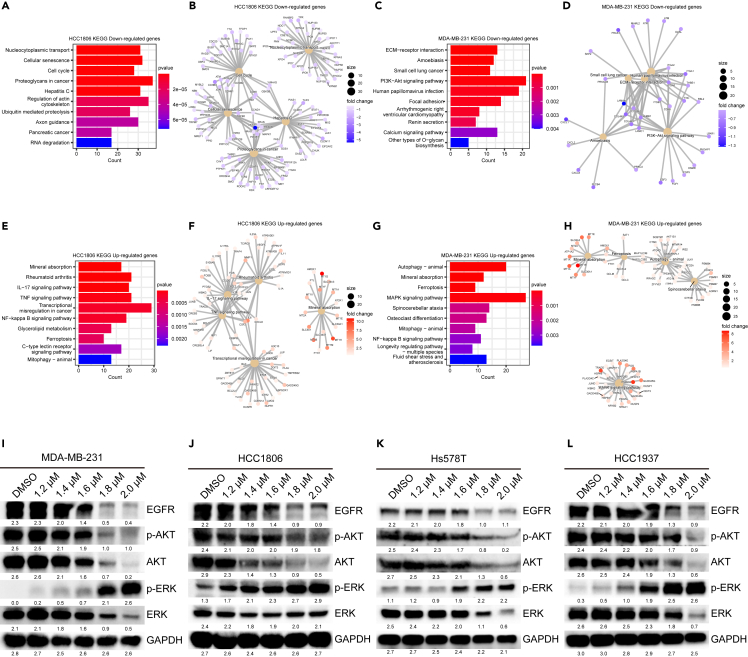
ZnPT downregulates EGFR-PI3K-AKT signaling pathway and upregulates MAPK signaling pathway (A–D) KEGG enrichment analysis of downregulated genes in ZnPT-treated TNBC cells. (E–H) KEGG enrichment analysis of upregulated genes in ZnPT-treated TNBC cells. (I–L) Western blot analysis of EGFR, *p*-AKT, AKT, *p*-ERK, and ERK proteins in ZnPT-treated TNBC cells. Data were presented as means ± SD. Two-tailed t test.

To investigate the intracellular signaling pathways affected by ZnPT, we conducted Kyoto Encyclopedia of Genes and Genomes (KEGG) enrichment analysis^71^ using previously obtained transcriptomic data. Our analysis demonstrated significant enrichment in downregulated genes within the PI3K-AKT signaling pathway (Figures 5C and 5D). The PI3K-AKT signaling pathway is frequently activated in various types of solid tumors, playing a dominant role in crucial aspects of carcinoma, such as cell proliferation, stemness, migration, and chemoresistance.^72^^,^^73^ Our results demonstrated that AKT3 gene expression was significantly downregulated in the PI3K-AKT signaling pathway (Figures 5A and 5B). To further explore whether ZnPT regulates AKT and its phosphorylation, we conducted western blot analysis of ZnPT-treated cell extracts. Results indicated inactivation of AKT phosphorylation and a reduction in total AKT protein levels (Figures 5I–5L). Carcinogenic activation of the PI3K-AKT signaling pathway can occur in TNBC due to the overexpression of upstream regulators, such as EGFR.^74^ Consistently, we also observed a reduction in EGFR protein levels upon ZnPT treatment (Figures 5I–5L).

Previous studies have highlighted that intracellular copper ions can stimulate the activation of the MAPK signaling pathway.^75^^,^^76^^,^^77^ Correspondingly, KEGG pathway enrichment analysis demonstrated that the mitogen-activated protein kinase (MAPK) signaling pathway was markedly upregulated under ZnPT treatment (Figures 5E–5H). Additionally, we observed a significant increase in the activation of phosphorylated ERK with increasing ZnPT concentrations (Figures 5I–5L). The novel copper complex HYF127c/Cu was previously shown to activate the MAPK signaling pathway by inducing reactive oxygen species (ROS), ultimately promoting autophagy.^78^ Consistently, KEGG analysis showed a significant upregulation in mitophagy, a form of autophagy that selectively degrades mitochondria (Figures 5E–5H). These findings suggest that ZnPT-induced enhancement of intracellular copper ions can lead to the oligomerization of mitochondrial proteins, such as DLAT, ultimately triggering proteotoxic stress and mitochondrial dysregulation. In conclusion, our results indicated that ZnPT downregulates AKT expression, thereby inhibiting the PI3K-AKT signaling pathway, while concurrently enhancing the MAPK signaling pathway and mitophagy by elevating intracellular copper ion levels.

## Discussion

Although ZnPT has a long application history as an antifungal agent, its exact cytotoxic mechanisms remain underexplored. However, recent studies have highlighted its promising antitumor capabilities. For instance, Forcina et al. (2017) used two human tumor cell lines to assess the impacts of over 1,800 bioactive compounds on cell death dynamics at the population level, identifying 13 compounds that rapidly triggered cell death, including ZnPT.^79^ In our study, ZnPT ranked third in potency among the 638 FDA-approved compounds tested against the MDA-MB-231 cell line. Based on detailed analyses of four TNBC cell lines, our results also indicated that ZnPT had significant inhibitory effects on cell proliferation, stemness, migration, and invasion at concentrations as low as 2 μM. Our xenograft experiments on nude mice further demonstrated the excellent therapeutic effects of ZnPT, with no discernible impact on mouse weight or blood, liver, and kidney function. In addition, our data revealed that ZnPT significantly inhibited multiple aberrantly activated signaling pathways in breast cancer, including EGFR, AKT, and STAT3. Taken together, our findings highlight the excellent potential of ZnPT as an effective agent against cancer cells.

Within cells, copper serves a dual function: while it acts as an essential cofactor for numerous proteins, excess copper can also induce stress and promote cell death. The cell permeability of simple copper salts is poor.^80^ In the cell membrane, SLC31A1 facilitates copper uptake, whereas ATP7A and ATP7B regulate its efflux, thus serving as copper transporters integral to copper trafficking.^81^^,^^82^^,^^83^ As a known ionophore, pyrithione also aids metal ion transport across cell membranes.^84^ In this study, direct ICP-MS evidence and indirect bioinformatics evidence indicated an elevated intracellular copper ion concentration with ZnPT treatment.

Cuproptosis primarily occurs within mitochondria, involving processes such as DLAT protein oligomerization and iron-sulfur protein downregulation, which ultimately trigger acute proteotoxic stress, as evidenced by elevated HSP70 protein levels.^7^ Rudolf and Cervinka (2010) observed the accumulation of zinc in mitochondria and disturbance of mitochondrial membrane potential in cervical tumor Hep-2 cells upon ZnPT treatment.^39^ Reeder et al. (2011) demonstrated the inactivation of iron-sulfur proteins in yeast following ZnPT exposure,^18^ while Justiniano et al. (2017) confirmed the upregulation of proteotoxic stress markers, including HSP70, in SCC-25 cells upon ZnPT treatment.^25^ These findings collectively support the potential of ZnPT at inducing cuproptosis in cancer cells. Consistent with these results, our data also revealed dysregulation of mitochondrial and iron-sulfur proteins, such as NAD(P)H, in TNBC cells. The confocal and native-PAGE analyses of DLAT further validated the oligomerization of DLAT, a hallmark feature of cuproptosis. In conclusion, based on the screening of various small molecules, we identified ZnPT as a potent inhibitor of TNBC and deciphered its underlying molecular mechanism. The observed effects of ZnPT closely align with the distinct characteristics of cuproptosis.

The mechanism of cuproptosis is distinct from that of apoptosis, which is dependent on caspase3 activation. Notably, our results demonstrated that treatment with ZnPT for 24 h did not lead to the upregulation of cleaved caspase3. Similarly, Forcina et al. (2017) demonstrated that ZnPT-induced cell death is not modulated by BAX and BAK inhibition or treatment with the pan-caspase inhibitor Q-VD-OPh.^79^ However, other studies have shown that ZnPT can induce apoptosis.^39^ This discrepancy may be due to the high ZnPT treatment concentration used in the experiments or the concurrent use of zinc and copper ions. Furthermore, as a non-specific copper ionophore, ZnPT may also promote the accumulation of intracellular iron levels. Indeed, our KEGG enrichment analysis of transcriptomic data showed that ferroptosis was significantly upregulated. These findings imply that ZnPT may modulate cell death depending on the presence of different metal ions. Considering the capacity of ZnPT to induce cell death via metal ions and the distinct characteristics of metal ion transport and binding observed in breast cancer, especially in TNBC, targeted therapies and diagnostics involving cuproptosis may be a unique and promising approach for effective TNBC treatment.

In conclusion, our study demonstrated that ZnPT dysregulates copper homeostasis and inhibits cell viability and proliferation in TNBC cells. Remarkably, ZnPT promotes the aggregation of DLAT, a biomarker of cuproptosis, both *in vitro* and *in vivo*. Furthermore, ZnPT suppresses the migration, invasion, and stemness in TNBC cells and inhibits TNBC development by downregulating the EGFR-PI3K-AKT signaling pathway and upregulating the MAPK signaling pathway. Collectively, this study revealed that ZnPT induces cell death by targeting copper homeostasis, providing an experimental basis for exploring cuproptosis as a potential target in drug discovery for TNBC patients.

### Limitations of the study

This study has several limitations. First, although we identified that ZnPT targeted cuproptosis to induce DLAT aggregation, we did not demonstrate the condition under which oligomerization occurred or the presence of lipoylated DLAT. Second, our study lacks relevant evidence concerning the interactions between copper ions and DLAT that initiate the lipoylation of DLAT. Third, our research only used the TCGA data and *in vitro* methods to speculate that TNBC is sensitive to cuproptosis, lacking experimental data in mice and patients. Additionally, our research lacks direct evidence how PI3K-AKT pathway and mitochondrial autophagy contributed to the cuproptosis. Despite these limitations, our research extends our understanding for future clinical trials on cuproptosis as a targeted approach for TNBC.

## STAR★Methods

### Key resources table

**Table:** 

REAGENT or RESOURCE	SOURCE	IDENTIFIER
**Antibodies**		

Cleaved caspase3	Cell Signaling Technology	Cat#9661S; RRID: AB_2341188
CD44	Cell Signaling Technology	Cat#37259S; RRID: AB_2750879
EGFR	ABclonal	Cat# A11351; RRID: AB_2861549
*p*-AKT	Cell Signaling Technology	Cat#4060S; RRID: AB_2315049
AKT	Cell Signaling Technology	Cat#4691S; RRID: AB_915783
p-MAPK	Cell Signaling Technology	Cat#4370T; RRID: AB_2315112
MAPK	Cell Signaling Technology	Cat#4695T; RRID: AB_390779
p-STAT3	Cell Signaling Technology	Cat#9145S; RRID: AB_2491009
STAT3	Cell Signaling Technology	Cat#9139S; RRID: AB_331757
Fibronectin	Sigma-Aldrich	Cat#F7387; RRID: AB_476988
Vimentin	Cell Signaling Technology	Cat#D21H3; RRID: AB_2797632
GAPDH	Bioworld	Cat#AP0063; RRID: AB_2651132
Ki67	Abcam	Cat#ab15580; RRID: AB_443209
DLAT	Cell Signaling Technology	Cat#12362S; RRID: AB_2797893
ACO2	Abcam	Cat#ab129069; RRID: AB_11144142
SDHB	Abcam	Cat#ab178423; RRID: AB_2861366

**Chemicals, peptides, and recombinant proteins**		

PRMI 1640 medium	Gibco	Cat#11875093
DMEM/F-12 medium	Gibco	Cat#11330032
DAPI	Vector Laboratories	Cat# H-1200
RIPA	Shanghai Yuanye Biotechnology	Cat# R21237
Protease inhibitor mixture	BioTools	Cat# B14001
Zinc pyrithione	APExBIO	Cat# B2201

**Critical commercial assays**		

CellTiter 96® AQueous One Solution Cell Proliferation Assay (MTS)	Promega	Cat#G3581
ALDEFLUOR™ Kit	Stem Cell Technologies	Cat#1700
BD Pharmingen™ FITC Annexin V Apoptosis Detection Kit I	BD Biosciences	Cat#556547

**Deposited data**		

Raw and analyzed data	GSA	HRA005642

**Experimental models: Cell lines**		

HCC1806	ATCC	Cat#CRL-2335
HCC1937	ATCC	Cat#CRL-2336
MDA-MB-231	ATCC	Cat#HTB-26
MDA-MB-468	ATCC	Cat#HTB-132
Hs578T	ATCC	Cat#HTB-126
BT549	ATCC	Cat#HTB-122

**Experimental models: Organisms/strains**		

Mouse: BALB/c naked mouse, female	Shanghai Model Organisms Center, Inc.	NA

**Software and algorithms**		

ImageJ	NIH	https://imagej.nih.gov/ij/index.html
GraphPad Prism	GraphPad	https://www.graphpad.com/
FlowJo	FlowJo	https://www.flowjo.com/
R v4.2.1	Open source	https://www.rproject.org/
R Studio	Open source	https://www.rstudio.com/

### Resource availability

#### Lead contact

Further information and requests for resources and reagents should be directed to and will be fulfilled by the lead contact, Baowei Jiao (jiaobaowei@mail.kiz.ac.cn).

#### Materials availability

This study did not generate any new reagents and all materials in this study are commercially available.

### Experimental model and study participant details

#### Cell culture

All cell lines were derived from the American Type Culture Collection (ATCC) and maintained at passage under standard culture conditions (37°C, 5% CO_2_) in culture medium with 1% penicillin/streptomycin solution. The human breast cancer cell lines HCC1806, HCC1937, and MDA-MB-468 were grown in Roswell Park Memorial Institute (PRMI) 1640 medium (11875093, Gibco) with 10% fetal bovine serum (FBS). The Hs578T cells were grown in PRMI 1640 medium with 10% FBS and 0.01 mg/mL insulin. The BT549 cells were grown in PRMI 1640 medium with 10% FBS and 0.023 U/mL insulin. The MDA-MB-231 cells were cultured in Dulbecco’s Modified Eagle Medium/Nutrient Mixture F-12 (DMEM/F-12, 11330032, Gibco) with 10% FBS.

#### Tumor xenografts in nude mice

All animal experiments were approved by the Institutional Animal Care and Use Committee, Kunming Institute of Zoology, Chinese Academy of Sciences. The HCC1806 cells were resuspended (2 × 10^5^) in phosphate-buffered saline (PBS)/Matrigel mixture (100 μL), then injected orthotopically into 8-week-old female nude mice. Each group of mice with transplanted tumors was then randomly assigned into two groups. Each experimental group contained three mice. Tumor size was measured with digital calipers every four days and calculated based on the following formula: 0.52 × length × (width)^2^. When tumors reach a size of at least 0.1 × 0.1 cm, intraperitoneal injections of ZnPT (5 mg/kg) or corn oil were initiated every four days. In accordance with institutional guidelines, all mice were housed in individual ventilated cages. During the treatment program, tumor sizes and body weights were measured every four days. After 40 days, all mice were sacrificed by cervical dislocation, and the tumors were collected and weighed.

### Method details

#### Cell viability assay

Cells were seeded in 96-well plates at a concentration of 8 000 cells/well with complete medium overnight, then treated with respective reagents for two days. To test the efficacy of drugs, concentrations were varied in the medium. Cell viability was then measured using the 3-(4,5-dimethylthiazol-2-yl)-5-(3-carboxymethoxyphenyl)-2-(4-sulfophenyl)-2H-tetrazolium (MTS) assay with CellTiter 96 AQueous One Solution Reagent (Promega) at 490 nm.

#### Cell proliferation assay

Cells were seeded in 96-well plates at a concentration of 2 000 cells/well with complete medium overnight, then treated with serial dilutions of compounds for five days. Cell proliferation was then measured using the MTS assay every day with CellTiter 96 AQueous One Solution Reagent (Promega) at 490 nm.

#### Immunofluorescence

Cells were seeded on coverslips in six-well plates (50 000 cells/well) with complete medium overnight, then treated with serial dilutions of compounds for two days. Cells were then fixed in 4% paraformaldehyde (PFA) and blocked with 0.1% TritonX-100 in 5% goat serum for 1 h, followed by incubation with primary antibodies against DLAT (12362S, CST, 1:100) overnight at 4°C. After washing three times with PBS, fluorescein-labeled (KPL, 1:200) and Alexa Fluor 555-labeled secondary antibodies (Life Technologies, 1:2 000) were used to treat cells for 1 h. Finally, after washing three times with PBS, the coverslips were mounted with 4’,6-diamidino-2-phenylindole (DAPI) (H-1200, Vector Laboratories) and observed via laser-scanning confocal microscopy (Nikon). The fluorescence counting function in ImageJ was then used to count the number of DLAT foci.

#### Analysis of foci number

Using ImageJ, the images (Select File→Open Samples→image.file) were converted to grayscale (Image→Type→8-bit) and the threshold was set using the automated routine (Process→Binary→Make Binary). After analyzing the Particles (Analyze→Analyze Particles), a particle count summary was shown in another data window.

#### Immunohistochemistry

Tumor tissues were fixed and embedded with paraffin for one week. Paraffin sections were deparaffinized and rehydrated in a series of degraded alcohols. Antigen retrieval was performed using 10 mM citrate buffer in a microwave for 20 min. Sections were incubated in 3% H_2_O_2_ for 15 min to inactivate endogenous peroxidase. The samples were blocked with 10% goat serum for 2 h and incubated with primary antibodies against Ki67 (ab15580, Abcam, 1:100) overnight at 4°C. After washing three times with PBS, the samples were incubated with horseradish peroxidase (HRP)-conjugated secondary antibody (Sigma, 1:200) at room temperature for 1 h. Finally, after again washing three times with PBS, the slides were stained with 3,3-diaminobenzidine (DAB) and countered with hematoxylin.

#### Western blotting

Cells were seeded in 10-cm plates (1 × 10^6^ cells) and cultured for 24 h, then treated with serial dilutions of compounds for two days. The cells were washed with PBS and lysed using radioimmunoprecipitation assay lysis buffer (R21237, Shanghai Yuanye Biotechnology) with a protease inhibitor mixture (B14001, BioTools, USA) on ice for 30 min. The lysed samples were separated using sodium dodecylsulphate-polyacrylamide gel electrophoresis (SDS-PAGE), then transferred onto polyvinylidene fluoride membranes and blocked with 5% nonfat dried milk for 1 h. The membranes were incubated with the indicated primary antibodies at 4°C overnight, washed three times with PBS, and incubated with HRP-conjugated secondary antibodies at room temperature for 1 h. After again washing three times with PBS, the samples were detected using a chemiluminescent HRP substrate (Millipore, USA) and quantification was performed via densitometry using ImageJ. The antibodies used for immunoblotting included: cleaved caspase3 (9661S; CST, 1:1 000), CD44 (37259S, CST, 1:1 000), EGFR (A11351, ABclonal, 1:1 000), p-AKT (4060S, CST, 1:1 000), AKT (4691S, CST, 1:1 000), p-MAPK (4370T, CST, 1:1 000), MAPK (4695T, CST, 1:1 000), p-STAT3 (9145S, CST, 1:1 000), STAT3 (9139S, CST, 1:1 000), fibronectin (F7387, Sigma, 1:1 000), vimentin (D21H3, CST, 1:1 000), GAPDH (AP0063, Bioworld, 1:5 000), and DLAT (12362S, CST, 1:1 000), ACO2 (ab129069, abcom, 1:1 000), SDHB (ab178423, abcom, 1:1 000).

#### Flow cytometry

Cells were seeded in 10-cm plates (1 × 10^6^ cells) and cultured for 24 h, then treated with serial dilutions of compounds for two days. To examine the ratio of BCSCs, ALDH enzymatic activity was assessed using an ALDEFLUOR kit (1700, Stem Cell Technologies) according to the provided manual. Cell apoptosis was assayed by flow cytometry with a FITC Annexin V apoptosis detection kit I (556547; BD Biosciences) according to the manufacturer’s instructions.

#### Mammosphere assay

Cells were seeded in ultra-low attachment 96-well plates at 2 000 cells/well with EpiCult-B Basal Medium (Human) (Stem Cell Technologies, BC, Canada) and EpiCult-B Proliferation Supplement (Human) (Stem Cell Technologies, BC, Canada) with hydrocortisone (H811182, Macklin, China) and heparin (Stem Cell Technologies, BC, Canada). Mammosphere formation efficiency was calculated after 10–14 days.

#### Clonal formation assay

Cells were seeded in six-well plates (500 cells/well) and incubated with serial dilutions of compounds at 37°C for 2 weeks. The cells were sequentially fixed with 4% PFA for 10 min and the clones were stained with 0.2% crystal violet.

#### Wound healing assay

Cells were seeded in six-well plates in confluent monolayers. Scratch wounds were created using a sterilized tip of pipette in serum-free medium, followed by treatment with serial dilutions of compounds for one day. Images were captured at 0, 12, and 24 h after wounding.

#### Cell migration and invasion assay

Cells were placed in the top chamber of the Transwell (Corning) (1 × 10^5^ cells/well), then transferred to the bottom chamber containing complete medium. Cells were incubated with serial dilutions of compounds at 37°C for 24 h. For visualization, cells in the top well were removed by wiping the top of the membrane with cotton swabs. The membrane filter was then stained with 0.5% crystal violet and photographed. In the invasion assay, before seeding the cells, the inserts were first coated with Matrigel (BD) for 12 h, with the cells and Matrigel then removed using a cotton swab.

#### ICP-MS

Cells were seeded in 10-cm plates (1 × 10^6^ cells) and cultured for 24 h, then treated with serial dilutions of compounds for one day. The cells were collected and washed twice with PBS, then counted using an automated cell counter and transferred to a 15-mL centrifuge tube. The samples were lyophilized using a freeze dryer (CHRIST, 2–4 LDplus), then mixed with 200 μL of 65% nitric acid and left overnight. The samples were then diluted with ultrapure water to a final volume of 1 mL and prepared for subsequent mass spectrometry. Amounts of metals were measured using an Agilent 7900 ICP-QMS in low-resolution mode, taking natural isotope distribution into account. Sample introduction was achieved with a micro-nebulizer (MicroMist, 0.2 mL/min) through a Scott spray chamber. Isotopes were measured using a collision-reaction interface with helium gas (5 mL/min) to remove polyatomic interferences. Scandium and indium internal standards were injected after inline mixing with the samples to ensure no signal drift and matrix effects. A mix of certified standards was measured at concentrations spanning those of the samples to convert count measurements to concentrations in the solution. Values were normalized against cell number.

#### RNA-seq analysis

Raw sequence data were processed through the standard Illumina pipelines for base-calling and fastq file generation. Paired-end reads were mapped to the human genome primary assembly (GRCh38), and the Ensembl human gene annotation for GRCh38 genebuild was used to improve accuracy of the mapping with HISAT2 software. HTseq was used to assign sequence reads to genes. Differential expression analysis was performed with the Bioconductor DESeq2 package. GO, KEGG, and GSEA enrichment analyses were performed with the Bioconductor clusterProfiler package. Breast cancer patient data were acquired through Bioconductor TCGAbiolinks package.

#### Survival analysis

Kaplan-Meier Plotter (https://kmplot.com/analysis/) was used to conduct survival analysis. The survival type was set to OS. Significantly different survival was based on a log-rank test, with *p* < 0.05.

### Quantification and statistical analysis

All experimental results were from at least three independent replicates. Data are shown as mean ± standard deviation (SD). ns, not significant; ∗, *p* < 0.05; ∗∗, *p* < 0.01; ∗∗∗, *p* < 0.001; ∗∗∗∗, *p* < 0.0001. Statistical significance of differences between means was assessed by two-tailed *t-*test. All statistical analyses and data graphing were performed using FlowJo v10, GraphPad Prism 8.0, or R.

## Data Availability

• Data: The raw sequence data reported in this paper have been deposited in the Genome Sequence Archive (Genomics, Proteomics & Bioinformatics 2021) in National Genomics Data Center (Nucleic Acids Res 2022), China National Center for Bioinformation / Beijing Institute of Genomics, Chinese Academy of Sciences (GSA-Human: HRA005642) that are publicly accessible at https://ngdc.cncb.ac.cn/gsa-human. • Code: This paper does not report any original code. • Additional information: Any additional information required to reanalyze the data reported in this paper is available from the lead contact upon reasonable request.

## Supplemental information

Document S1. Figures S1–S6 and Data S1–S7

Table S1. List of 638 compounds screened in FDA-approved drug library, related to Figure 1
