# Supplementary material for: Targeting cuproptosis by zinc pyrithione in triple-negative breast cancer
Source: iScience. 2023 Oct 16;26(11):108218. doi: 10.1016/j.isci.2023.108218 (PMC10637938; doi:10.1016/j.isci.2023.108218)

iScience, Volume *26*

Supplemental information

Targeting cuproptosis by zinc

pyrithione in triple-negative breast cancer

Xu Yang, Li Deng, Xianhong Diao, Siyuan Yang, Li Zou, Qin Yang, Jian Li, Jianyun Nie, Lina Zhao, and Baowei Jiao

Supplemental legend

Figure S1. Screening of 638 compounds in FDA-approved drug library, Related to Figure 1.

(A-O) Cell viability testing of 638 compounds in MDA-MB-231 cells.

Figure S2. ZnPT induces copper ion disorder and represses cell proliferation in TNBC cells, Related to Figure 1.

(A-D) TNBC cell viability under napabucasin treatment. (E-H) TNBC cell viability under penfluridol treatment. (I-J) TNBC cell proliferation under ZnPT treatment. (K-R) GO enrichment analysis of up-regulated genes in ZnPT-treated TNBC cells. CC: Cell Component; MF: Molecular Function. (S-T) GSEA enrichment analysis of differentially expressed genes in ZnPT-treated TNBC cells. Data were presented as means ± SD. *, $p < 0.05$; **, $p < 0.01$; ***, $p < 0.001$; ****, $p < 0.0001$, two-tailed *t*-test.

Figure S3. RNA-seq analysis of TNBC cells following ZnPT treatment, Related to Figure 1.

(A, C) Volcano map showing overall differentially expressed mRNAs in ZnPT-treated TNBC cells. Each point represents an mRNA. Points in red indicate up-regulated genes, points in blue indicate down-regulated genes. Gray dots indicate non-differentially expressed genes. (B, D) Principal component analysis of total detected mRNAs in Z

and D groups. Z: ZnPT-treated; D: DMSO. (E-P) GO enrichment analysis of down-regulated genes in ZnPT-treated TNBC cells. BP: Biological Process; CC: Cell Component; MF: Molecular Function. (Q-R) GSEA enrichment analysis of differentially expressed genes in ZnPT-treated TNBC cells. Data were presented as means ± SD. Two-tailed *t*-test.

Figure S4. ZnPT promotes oligomerization of DLAT *in vitro* and shows no adverse effects *in vivo*, Related to Figure 2.

(A-B) Representative images of immunofluorescence in ZnPT-treated TNBC cells, scale bar 5μm. (C-D) Statistical analysis of DLAT foci number in ZnPT-treated TNBC cells. (E-F) Western blotting of ACO2 and SDHB proteins in ZnPT-treated TNBC cells. (G, I) Representative images of apoptosis cell ratios using flow cytometry. (H, J) Statistical analysis of early and late apoptosis cell ratios in ZnPT-treated TNBC cells. (K) Western blot analysis of cleaved caspase3 proteins in ZnPT-treated TNBC cells. (L) Representative immunohistochemical images of Ki67 in ZnPT-treated nude mice, scale bar 800μm. (M) Statistical analysis of Ki67+ cells in ZnPT-treated nude mice. (N-O) Native-PAGE of DLAT in ZnPT-treated TNBC cells. (P) Host body weight curve. (O-S) Statistical analysis of immune cell numbers in ZnPT-treated nude mice. WBC: white blood cell; RBC: red blood cell; PLT: platelet. (T-U) Statistical analysis of enzyme activity in ZnPT-treated nude mice. ALT: alanine aminotransferase; AST: aspartate aminotransferase. (V-W) Statistical analysis of metabolite concentration in ZnPT-

treated nude mice. BUN: blood urea nitrogen; CRE: creatinine. Data were presented as means ± SD. ns, not significant. Two-tailed *t*-test.

Figure S5. Bioinformatics analysis of genes related to cuproptosis signaling pathways, Related to Figure 3.

(A) Transcript expression levels of DLD and GCSH in normal breast tissue and different breast cancer subtypes in TCGA. (B) Kaplan-Meier survival analysis showing correlation between OS and DLD, DLST, and GCSH mRNA expression. (C) Kaplan-Meier survival analysis showing correlation between OS and DLD, DLST, and GCSH protein expression. (D-H) GO enrichment analysis of up-regulated genes in breast cancer patients with high DBT, DLD, GCSH, LIAS, and LIPT1 expression. (I-M) Analysis of TCGA dataset for expression correlation between DBT, DLD, DLST, LIPT1 and ALDH1A1, ALDH1A3, CD44, STAT3. (N-O) Representative images of ALDH+ cell ratios using flow cytometry. (P-Q) Statistical analysis of ALDH+ cell ratios in ZnPT-treated TNBC cells. (R) Western blot analysis of CD44, p-STAT3, and STAT3 proteins in ZnPT-treated TNBC cells. Data were presented as means ± SD. Two-tailed *t*-test.

Figure S6. ZnPT suppresses migration, invasion, and stemness in TNBC cells, Related to Figure 4.

(A) Representative images of clonal formation assays in ZnPT-treated TNBC cells, scale bar 1cm. (B) Statistical analysis of number of clones in ZnPT-treated TNBC cells.

(C) Representative images of tumorspheres in ZnPT-treated TNBC cells, scale bar 200μm. (D) Statistical test of number of tumorspheres in ZnPT-treated TNBC cells. (E) Statistical test of size of tumorspheres in ZnPT-treated TNBC cells. (F) Representative images of wound healing assays in ZnPT-treated TNBC cells, scale bar 400μm. (G) Statistical analysis of healing rate in ZnPT-treated TNBC cells. (H) Representative images of cell migration and invasion assays in ZnPT-treated TNBC cells, scale bar 10μm. (I) Statistical analysis of migration and invasion cell number in ZnPT-treated TNBC cells. (J) Western blot analysis of vimentin and fibronectin proteins in ZnPT-treated TNBC cells. FN1: Fibronectin; VIM: Vimentin. Data were presented as means ± SD. *, $p < 0.05$; **, $p < 0.01$; ***, $p < 0.001$; ****, $p < 0.0001$, two-tailed *t*-test.

Data S1. Raw western blots related to Figure 2, Related to Figure 2.

Data S2. Raw western blots related to Figure 3, Related to Figure 3.

Data S3. Raw western blots related to Figure 4, Related to Figure 4.

Data S4. Raw western blots related to Figure 5, Related to Figure 5.

Data S5. Raw western blots related to Figure S4, Related to Figure S4.

Data S6. Raw western blots related to Figure S5, Related to Figure S5.

Data S7. Raw western blots related to Figure S6, Related to Figure S6.

Fig S1

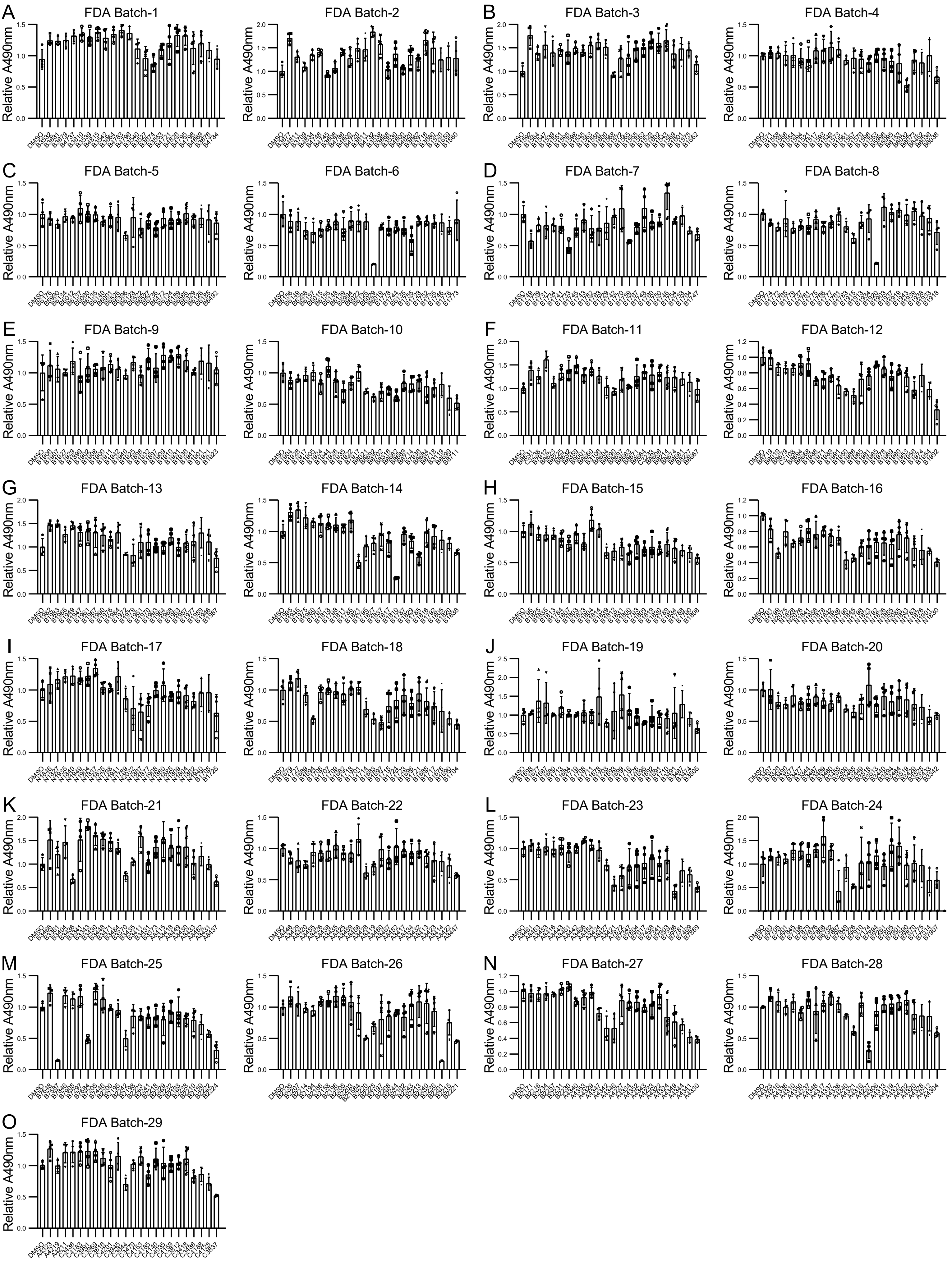

A
FDA Batch-1
FDA Batch-2
B
FDA Batch-3
FDA Batch-4
C
FDA Batch-5
FDA Batch-6
D
FDA Batch-7
FDA Batch-8
E
FDA Batch-9
FDA Batch-10
F
FDA Batch-11
FDA Batch-12
G
FDA Batch-13
FDA Batch-14
H
FDA Batch-15
FDA Batch-16
I
FDA Batch-17
FDA Batch-18
J
FDA Batch-19
FDA Batch-20
K
FDA Batch-21
FDA Batch-22
L
FDA Batch-23
FDA Batch-24
M
FDA Batch-25
FDA Batch-26
N
FDA Batch-27
FDA Batch-28
O
FDA Batch-29
Relative A490nm
DMSO

Fig S2

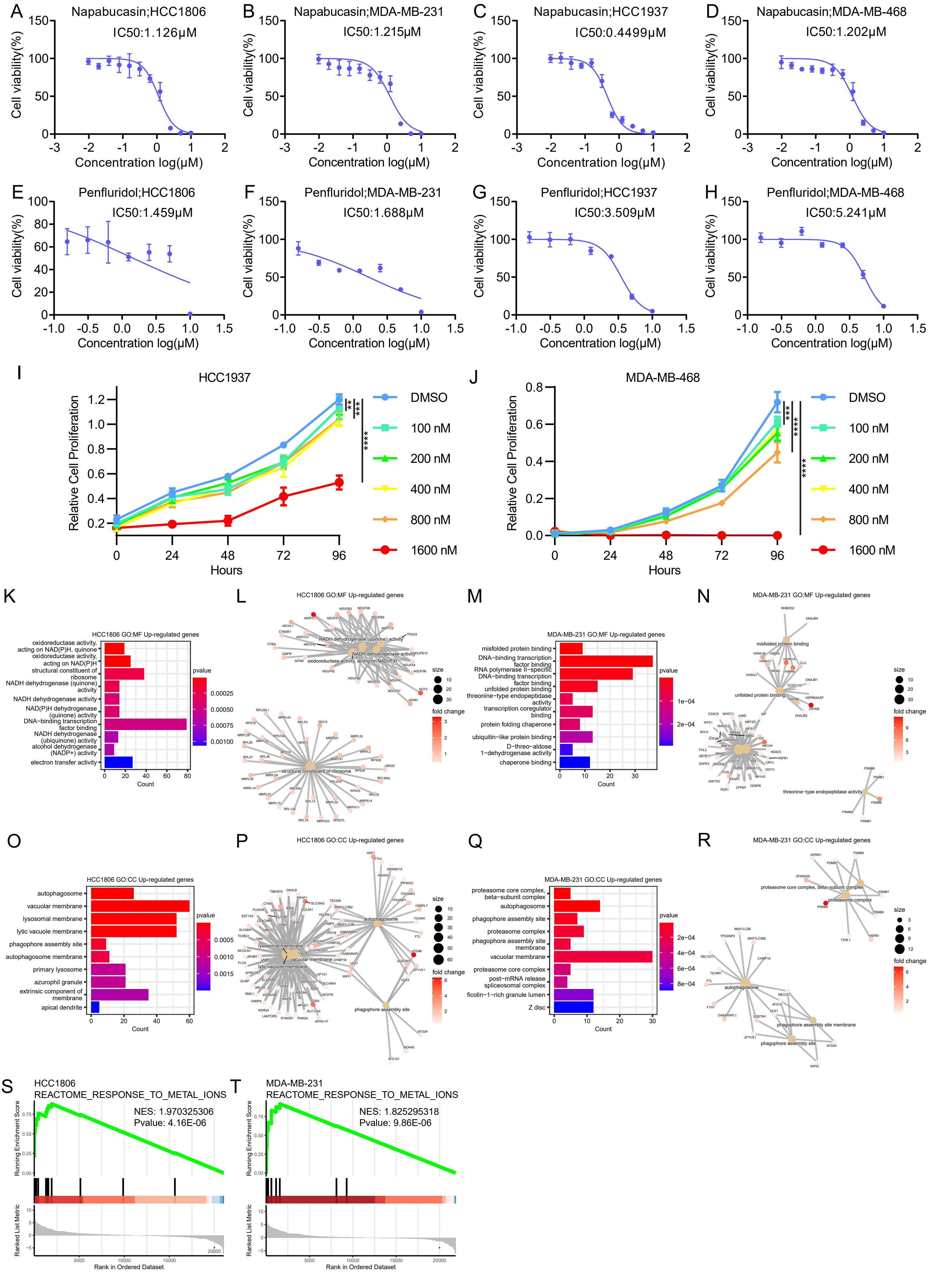
A
Napabucasin;HCC1806
IC50:1.126μM
B
Napabucasin;MDA-MB-231
IC50:1.215μM
C
Napabucasin;HCC1937
IC50:0.4499μM
D
Napabucasin;MDA-MB-468
IC50:1.202μM
Cell viability(%)
Concentration log(μM)
E
Penfluridol;HCC1806
IC50:1.459μM
F
Penfluridol;MDA-MB-231
IC50:1.688μM
G
Penfluridol;HCC1937
IC50:3.509μM
H
Penfluridol;MDA-MB-468
IC50:5.241μM
I
HCC1937
J
MDA-MB-468
Relative Cell Proliferation
Hours
DMSO
100 nM
200 nM
400 nM
800 nM
1600 nM
K
HCC1806 GO:MF Up-regulated genes
L
HCC1806 GO:MF Up-regulated genes
M
MDA-MB-231 GO:MF Up-regulated genes
N
MDA-MB-231 GO:MF Up-regulated genes
O
HCC1806 GO:CC Up-regulated genes
P
HCC1806 GO:CC Up-regulated genes
Q
MDA-MB-231 GO:CC Up-regulated genes
R
MDA-MB-231 GO:CC Up-regulated genes
S
HCC1806
REACTOME_RESPONSE_TO_METAL_IONS
NES: 1.970325306
Pvalue: 4.16E-06
T
MDA-MB-231
REACTOME_RESPONSE_TO_METAL_IONS
NES: 1.825295318
Pvalue: 9.86E-06
Running Enrichment Score
Ranked List Metric
Rank in Ordered Dataset

Fig S3

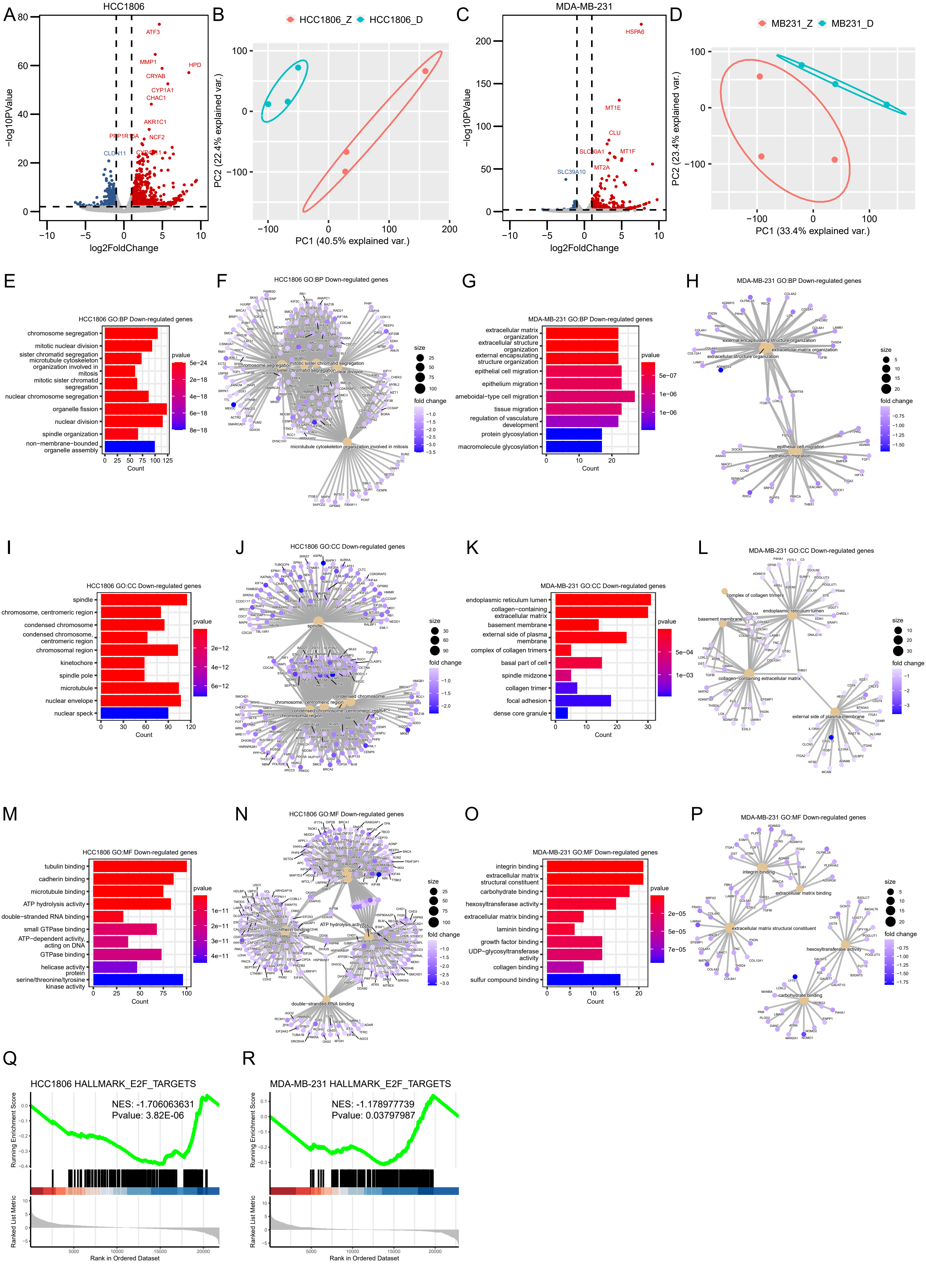

HCC1806
MDA-MB-231
log2FoldChange
-log10PValue
HCC1806_Z
HCC1806_D
MB231_Z
MB231_D
PC1 (40.5% explained var.)
PC2 (22.4% explained var.)
PC1 (33.4% explained var.)
PC2 (23.4% explained var.)
HCC1806 GO:BP Down-regulated genes
MDA-MB-231 GO:BP Down-regulated genes
HCC1806 GO:CC Down-regulated genes
MDA-MB-231 GO:CC Down-regulated genes
HCC1806 GO:MF Down-regulated genes
MDA-MB-231 GO:MF Down-regulated genes
HCC1806 HALLMARK_E2F_TARGETS
NES: -1.706063631
Pvalue: 3.82E-06
MDA-MB-231 HALLMARK_E2F_TARGETS
NES: -1.178977739
Pvalue: 0.03797987
Running Enrichment Score
Ranked List Metric
Rank in Ordered Dataset

Fig S4

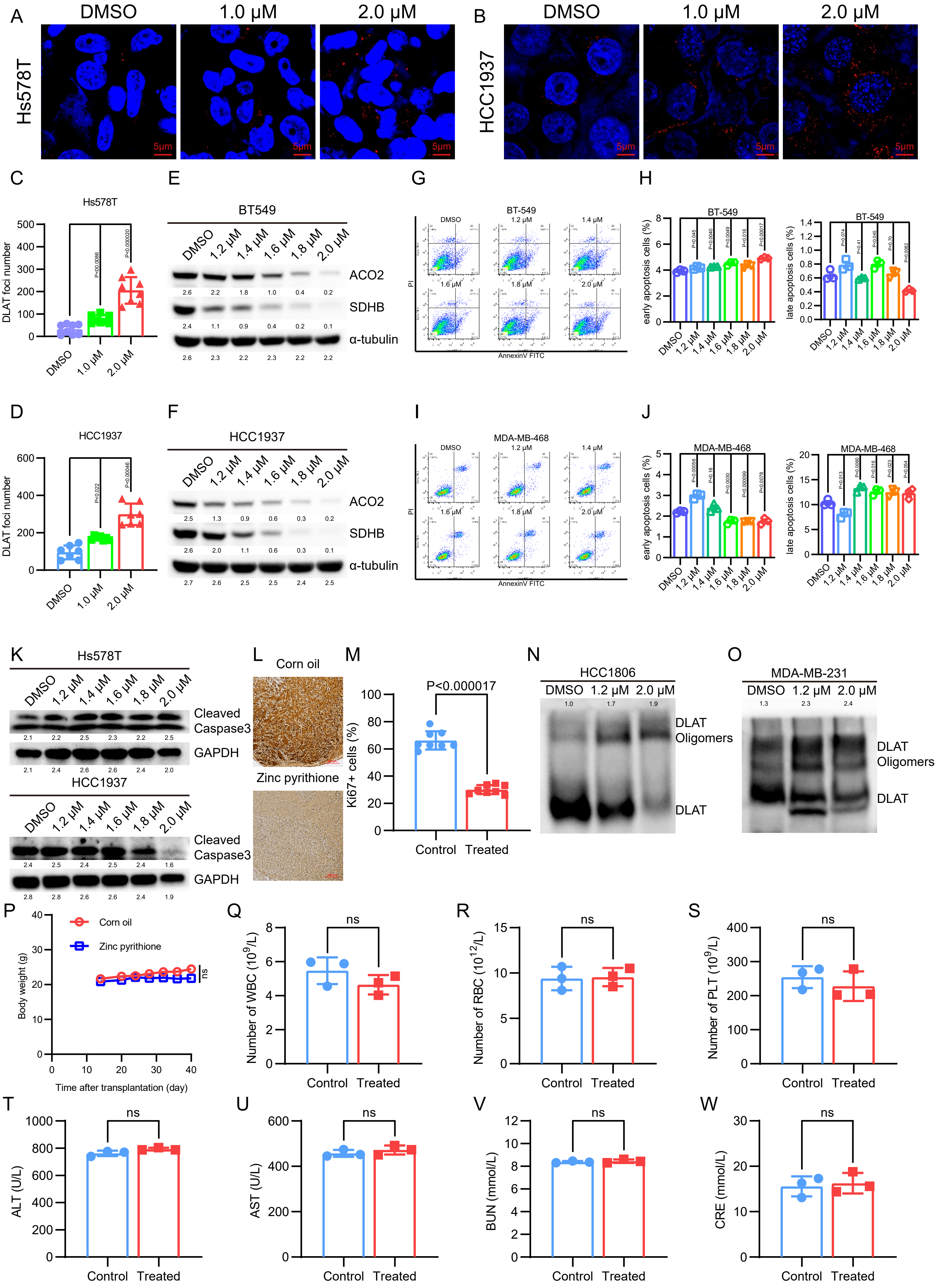
A
DMSO
1.0 μM
2.0 μM
Hs578T
5μm
B
HCC1937
C
Hs578T
DLAT foci number
DMSO
1.0 μM
2.0 μM
D
HCC1937
E
BT549
ACO2
SDHB
α-tubulin
F
HCC1937
G
BT-549
AnnexinV FITC
PI
H
early apoptosis cells (%)
late apoptosis cells (%)
I
MDA-MB-468
J
K
Hs578T
Cleaved Caspase3
GAPDH
L
Corn oil
Zinc pyrithione
M
P<0.000017
Ki67+ cells (%)
Control
Treated
N
HCC1806
DLAT Oligomers
DLAT
O
MDA-MB-231
P
Corn oil
Zinc pyrithione
Body weight (g)
Time after transplantation (day)
ns
Q
Number of WBC (10^9/L)
R
Number of RBC (10^12/L)
S
Number of PLT (10^9/L)
T
ALT (U/L)
U
AST (U/L)
V
BUN (mmol/L)
W
CRE (mmol/L)

Fig S5

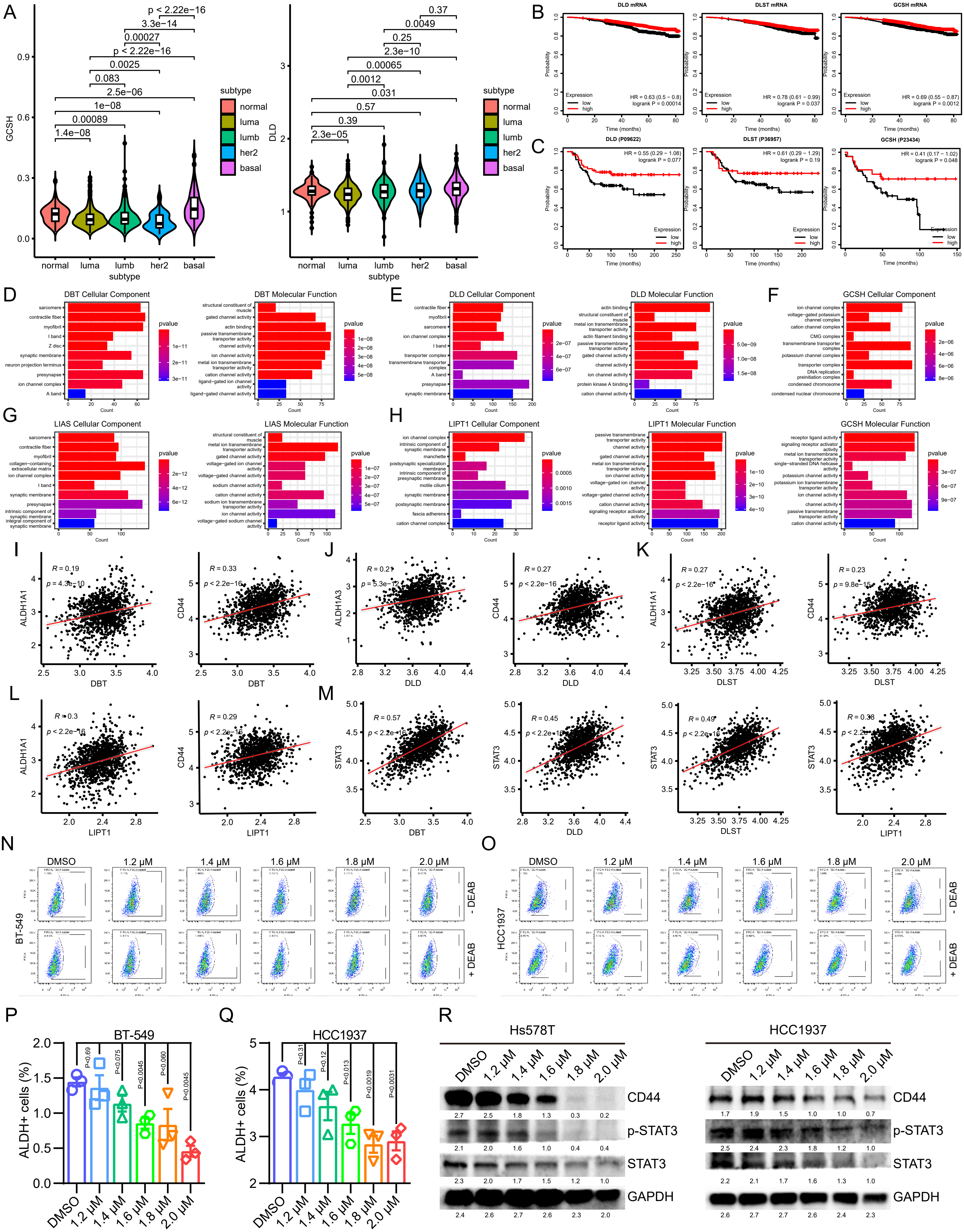
A
p < 2.22e−16
3.3e−14
0.00027
p < 2.22e−16
0.0025
0.083
2.5e−06
1e−08
0.00089
1.4e−08
GCSH
subtype
normal
luma
lumb
her2
basal
0.37
0.0049
0.25
2.3e−10
0.00065
0.0012
0.031
0.57
0.39
2.3e−05
DLD
B
DLD mRNA
DLST mRNA
GCSH mRNA
HR = 0.63 (0.5 − 0.8)
logrank P = 0.00014
HR = 0.78 (0.61 − 0.99)
logrank P = 0.037
HR = 0.69 (0.55 − 0.87)
logrank P = 0.0012
Expression
low
high
Time (months)
Probability
C
DLD (P09622)
DLST (P36957)
GCSH (P23434)
HR = 0.55 (0.29 − 1.08)
logrank P = 0.077
HR = 0.61 (0.29 − 1.29)
logrank P = 0.19
HR = 0.41 (0.17 − 1.02)
logrank P = 0.048
D
DBT Cellular Component
DBT Molecular Function
E
DLD Cellular Component
DLD Molecular Function
F
GCSH Cellular Component
G
LIAS Cellular Component
LIAS Molecular Function
H
LIPT1 Cellular Component
LIPT1 Molecular Function
GCSH Molecular Function
pvalue
Count
I
R = 0.19
p = 4.3e−10
R = 0.33
p < 2.2e−16
ALDH1A1
CD44
DBT
J
R = 0.21
p = 5.3e−12
R = 0.27
p < 2.2e−16
ALDH1A3
DLD
K
R = 0.27
p < 2.2e−16
R = 0.23
p = 9.8e−15
DLST
L
R = 0.3
p < 2.2e−16
R = 0.29
p < 2.2e−16
LIPT1
M
R = 0.57
p < 2.2e−16
R = 0.45
p < 2.2e−16
R = 0.49
p < 2.2e−16
R = 0.38
STAT3
N
DMSO
1.2 μM
1.4 μM
1.6 μM
1.8 μM
2.0 μM
BT-549
- DEAB
+ DEAB
O
HCC1937
P
BT-549
P<0.69
P<0.075
P<0.0045
P<0.060
P<0.0045
ALDH+ cells (%)
Q
HCC1937
P<0.31
P<0.12
P<0.013
P<0.0019
P<0.0031
R
Hs578T
CD44
2.7 2.5 1.8 1.3 0.3 0.2
p-STAT3
2.1 2.0 1.6 1.0 0.4 0.4
STAT3
2.3 2.0 1.7 1.5 1.2 1.0
GAPDH
2.4 2.6 2.7 2.6 2.3 2.0
HCC1937
CD44
1.7 1.9 1.5 1.0 1.0 0.7
p-STAT3
2.5 2.4 2.3 1.8 1.2 1.0
STAT3
2.2 2.1 1.7 1.6 1.3 1.0
GAPDH
2.6 2.7 2.7 2.6 2.4 2.3

Fig S6

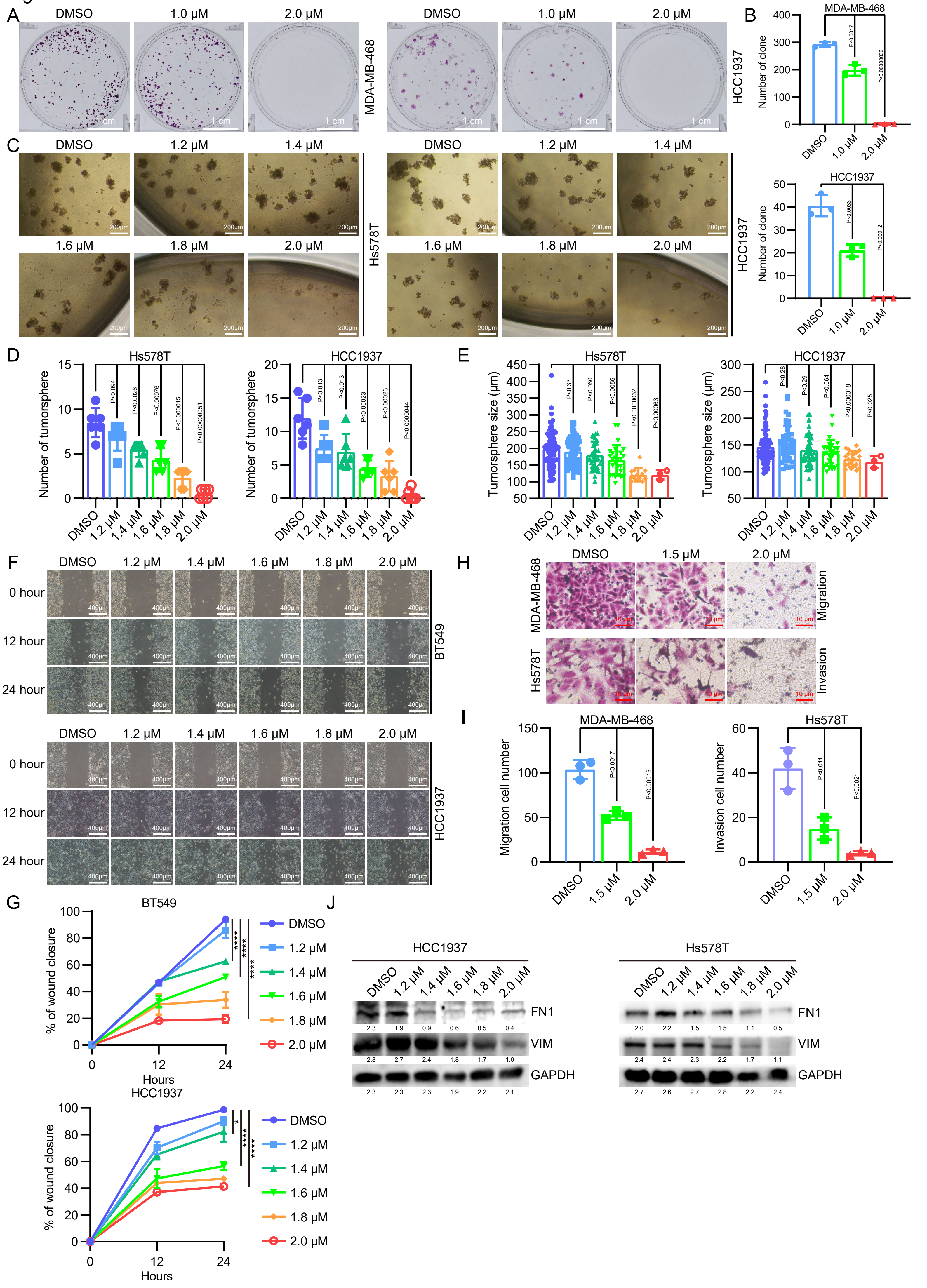

A
DMSO
1.0 μM
2.0 μM
MDA-MB-468
HCC1937
1 cm
B
MDA-MB-468
Number of clone
P<0.0017
P<0.00000032
HCC1937
P<0.0033
P<0.00012
DMSO
1.0 μM
2.0 μM
C
DMSO
1.2 μM
1.4 μM
1.6 μM
1.8 μM
2.0 μM
Hs578T
200μm
D
Hs578T
Number of tumorsphere
P<0.094
P<0.0026
P<0.00076
P<0.000015
P<0.00000051
HCC1937
P<0.013
P<0.013
P<0.00023
P<0.00023
P<0.0000044
E
Hs578T
Tumorsphere size (μm)
P<0.33
P<0.060
P<0.0056
P<0.0000032
P<0.00063
HCC1937
P<0.28
P<0.29
P<0.064
P<0.000018
P<0.025
F
0 hour
12 hour
24 hour
BT549
400μm
H
MDA-MB-468
Hs578T
DMSO
1.5 μM
2.0 μM
Migration
Invasion
10 μm
I
MDA-MB-468
Migration cell number
P<0.0017
P<0.00013
Hs578T
Invasion cell number
P<0.011
P<0.0021
G
BT549
% of wound closure
Hours
****
HCC1937
*
J
HCC1937
Hs578T
FN1
VIM
GAPDH
2.3 1.9 0.9 0.6 0.5 0.4
2.8 2.7 2.4 1.8 1.7 1.0
2.3 2.3 2.3 1.9 2.2 2.1
2.0 2.2 1.5 1.5 1.1 0.5
2.4 2.4 2.3 2.2 1.7 1.1
2.7 2.6 2.7 2.8 2.2 2.4

Data S1

MDA-MB-231
Cleaved Caspase3

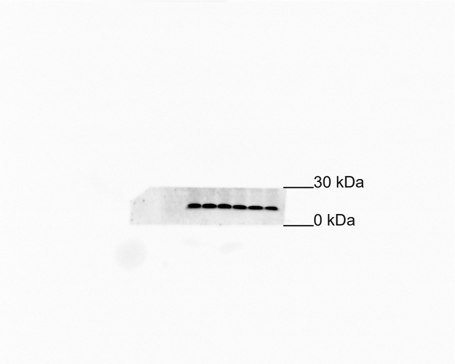

MDA-MB-231
GAPDH

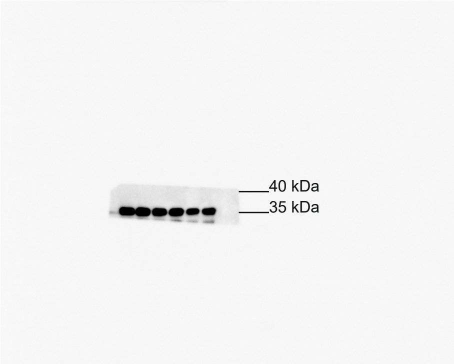

HCC1806
Cleaved Caspase3

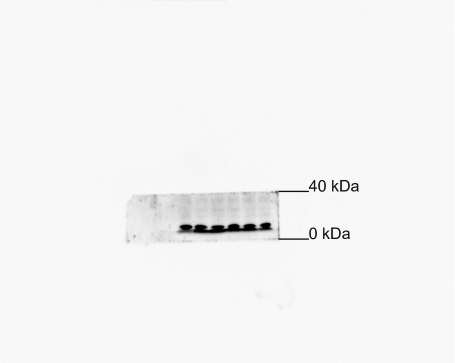

HCC1806
GAPDH

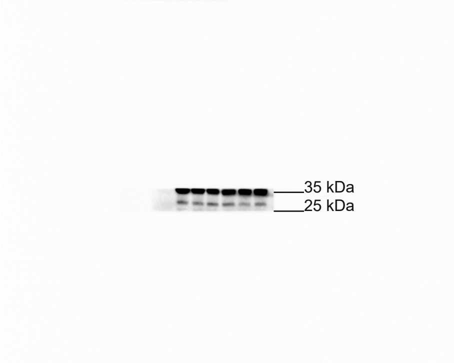

MDA-MB-231
ACO2

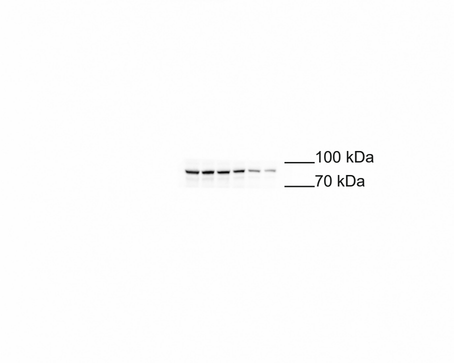

MDA-MB-231
SDHB

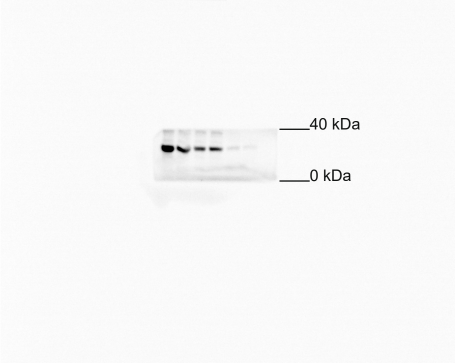

MDA-MB-231
α-tubulin

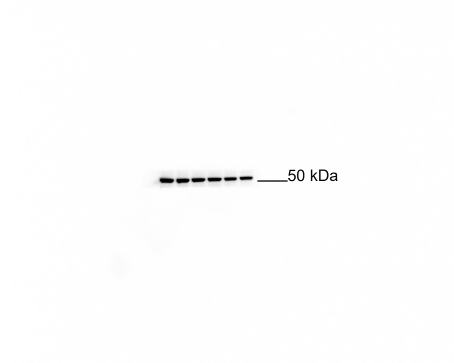

HCC1806
ACO2

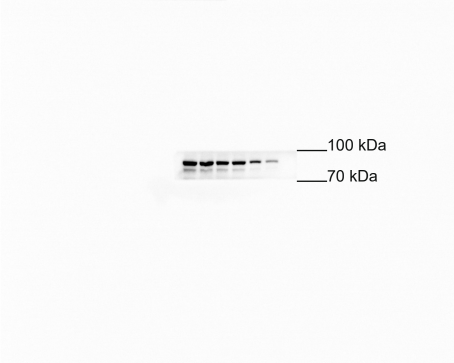

HCC1806
SDHB

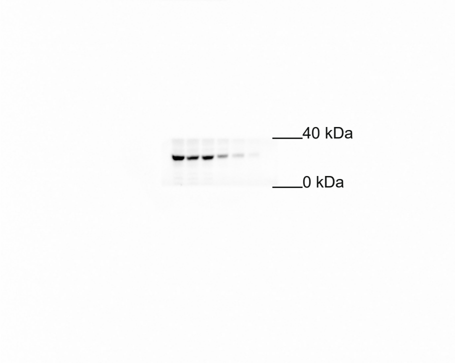

HCC1806
α-tubulin

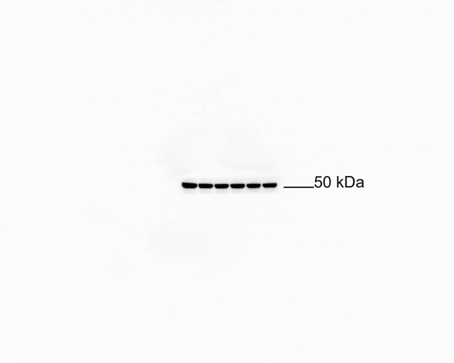

Data S2

MDA-MB-231
CD44

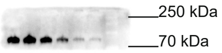

MDA-MB-231
p-STAT3

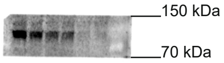

MDA-MB-231
STAT3

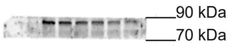

MDA-MB-231
GAPDH

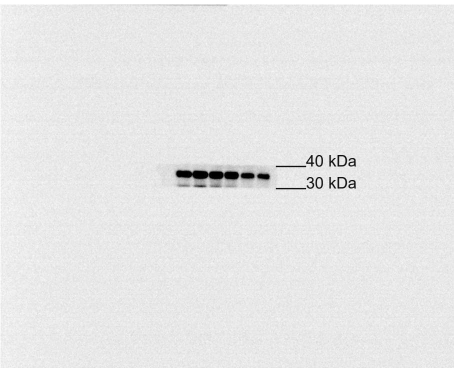

HCC1806
CD44

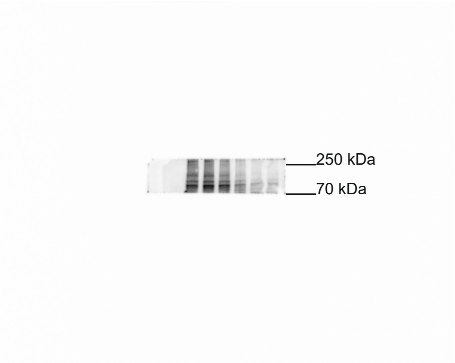

HCC1806
p-STAT3

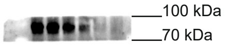

HCC1806
STAT3

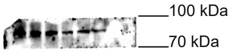

HCC1806
GAPDH

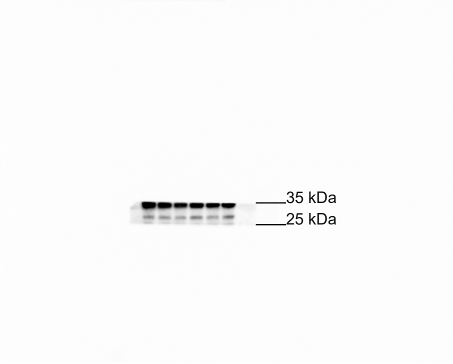

Data S3

MDA-MB-231
FN1

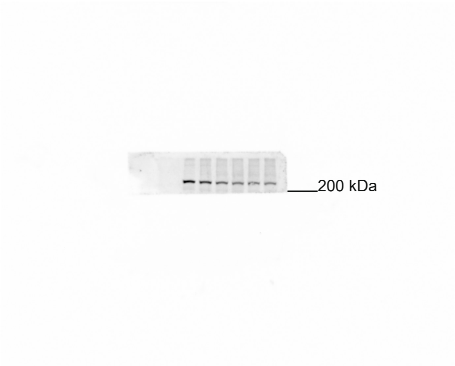

MDA-MB-231
VIM

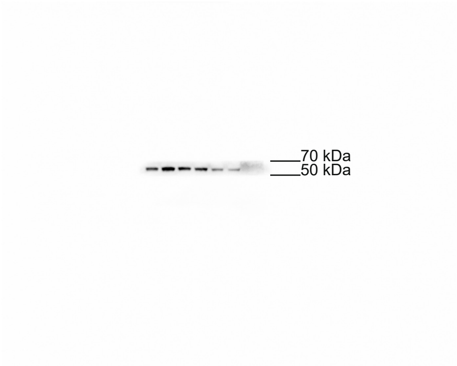

MDA-MB-231
GAPDH

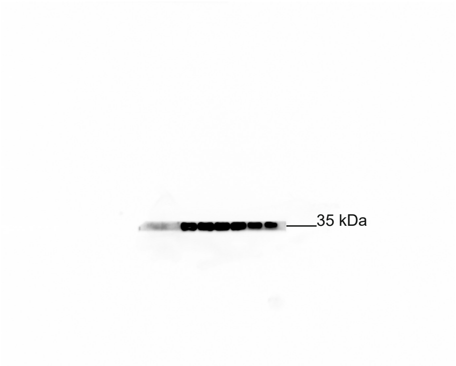

HCC1806
FN1

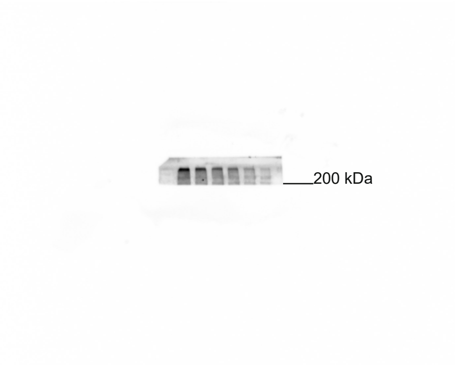

HCC1806
VIM

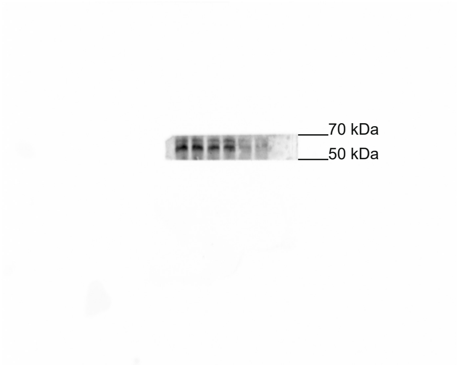

HCC1806
GAPDH

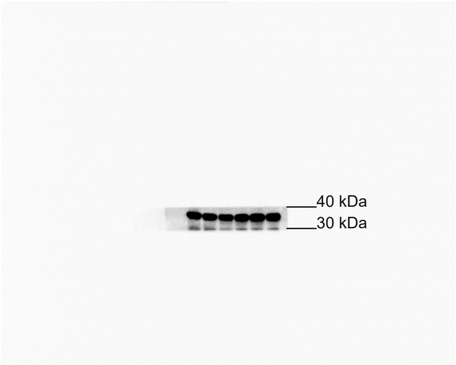

Data S4

MDA-MB-231
EGFR

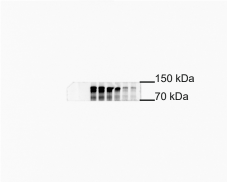

MDA-MB-231
p-AKT

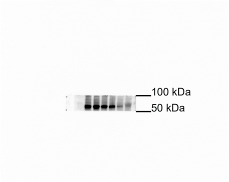

MDA-MB-231
AKT

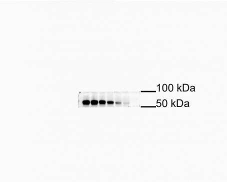

MDA-MB-231
p-ERK

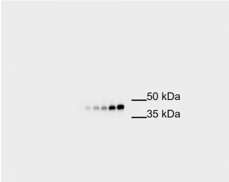

MDA-MB-231
ERK

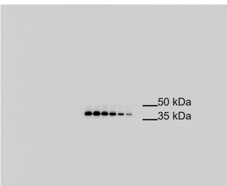

MDA-MB-231
GAPDH

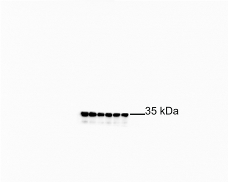

HCC1806
EGFR

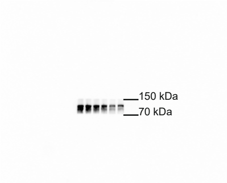

HCC1806
p-AKT

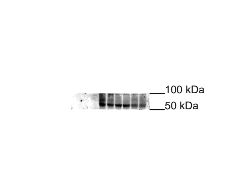

HCC1806
AKT

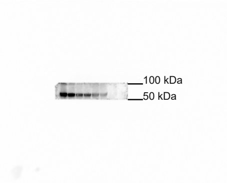

HCC1806
p-ERK

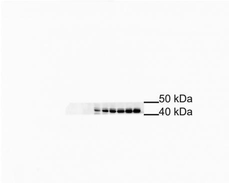

HCC1806
ERK

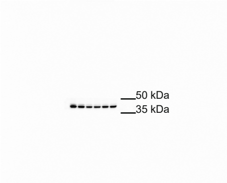

HCC1806
GAPDH

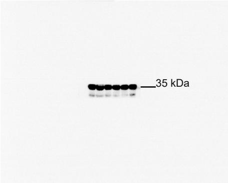

Hs578T
EGFR

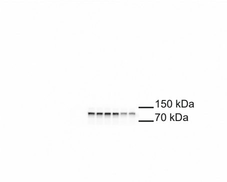

Hs578T
p-AKT

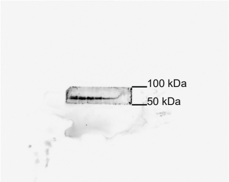

Hs578T
AKT

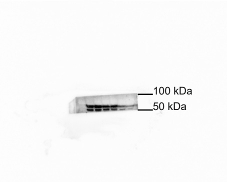

Hs578T
p-ERK

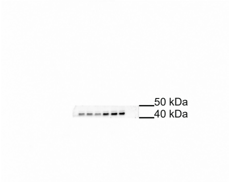

Hs578T
ERK

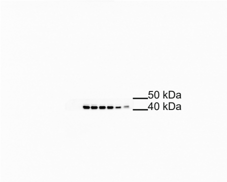

Hs578T
GAPDH

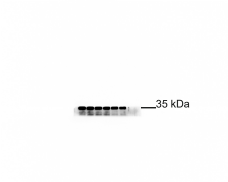

HCC1937
EGFR

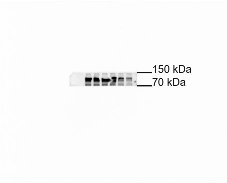

HCC1937
p-AKT

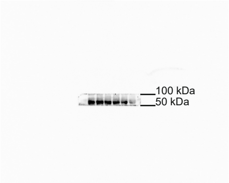

HCC1937
AKT

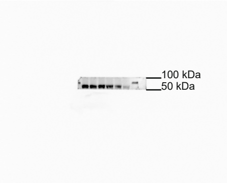

HCC1937
p-ERK

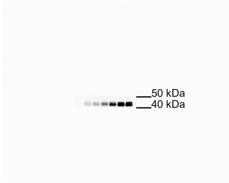

HCC1937
ERK

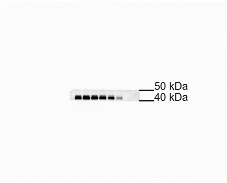

HCC1937
GAPDH

Data S5

Hs578T
Cleaved Caspase3

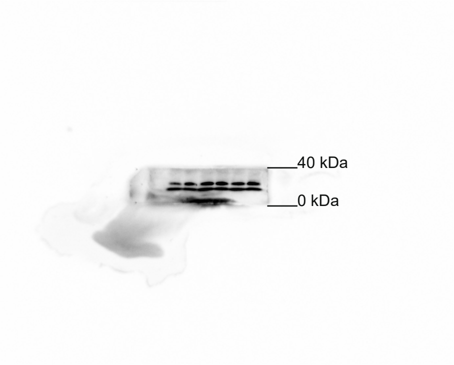

Hs578T
GAPDH

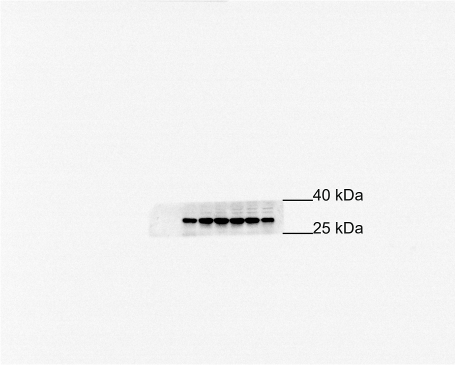

MDA-MB-231
DLAT

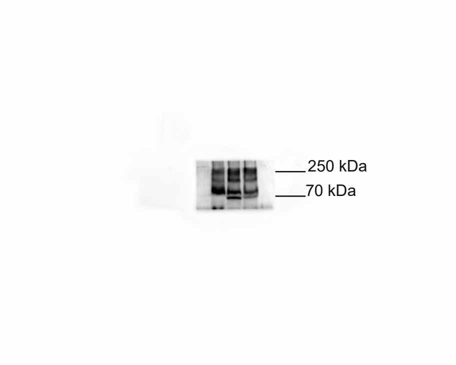

HCC1937
Cleaved Caspase3

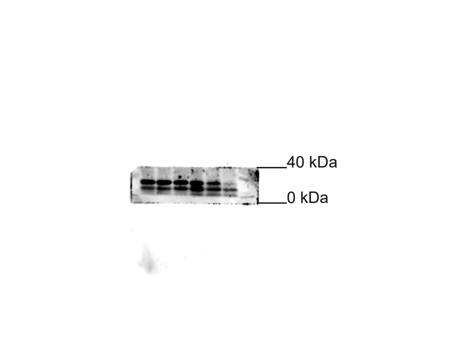

HCC1937
GAPDH

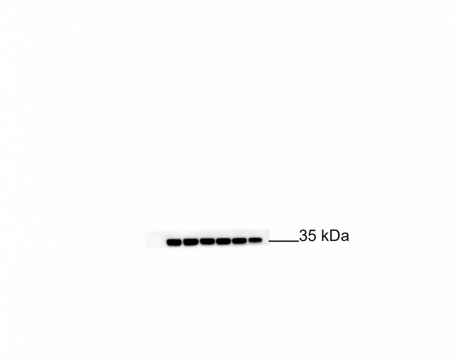

HCC1806
DLAT

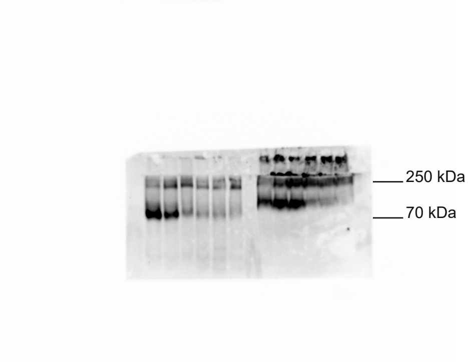

BT549
ACO2

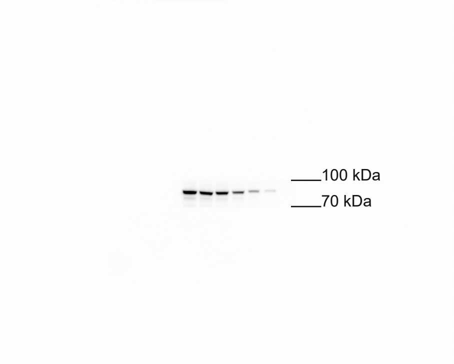

BT549
SDHB

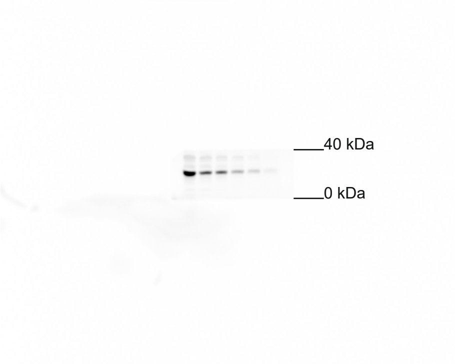

BT549
α-tubulin

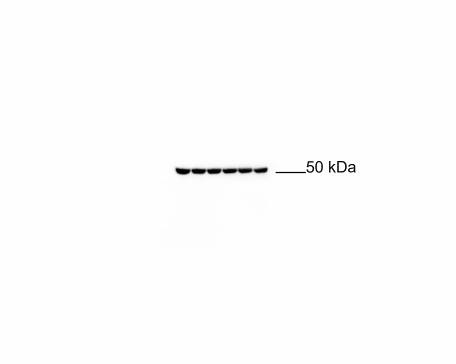

HCC1937
ACO2

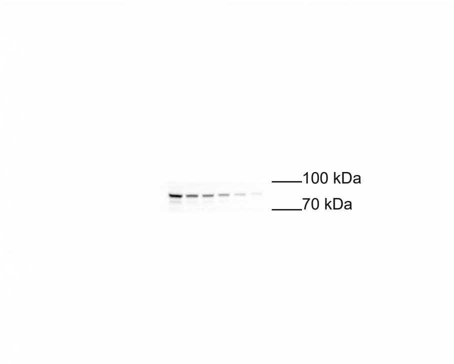

HCC1937
SDHB

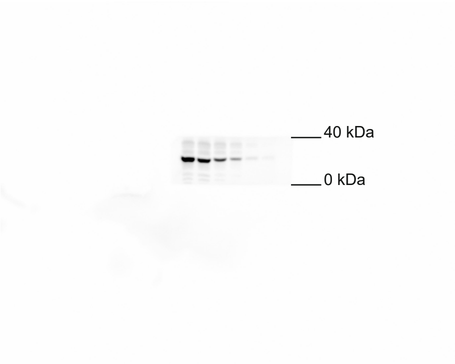

HCC1937
α-tubulin

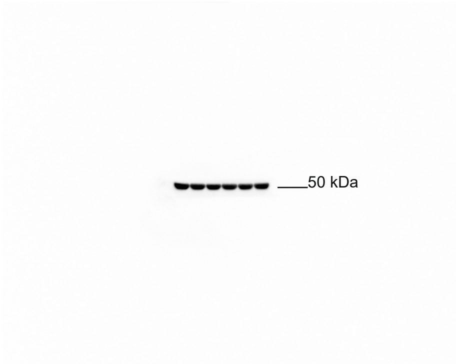

Data S6

Hs578T
CD44

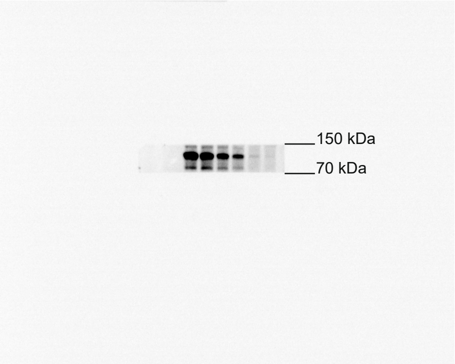

Hs578T
p-STAT3

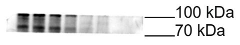

Hs578T
STAT3

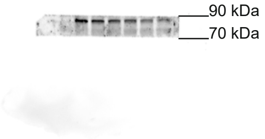

Hs578T
GAPDH

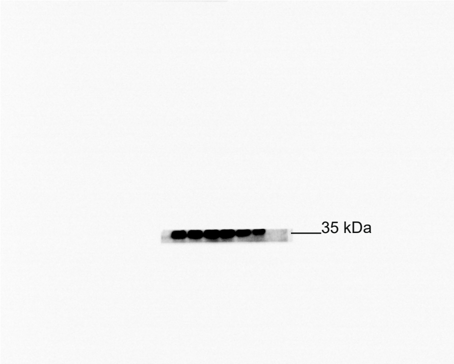

HCC1937
CD44

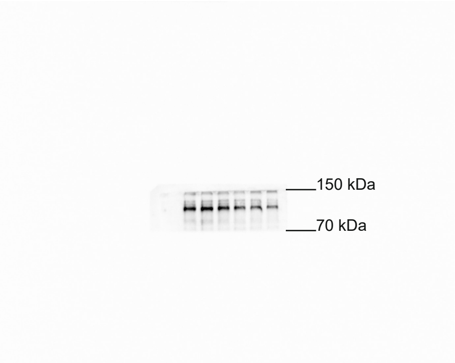

HCC1937
p-STAT3

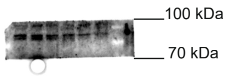

HCC1937
STAT3

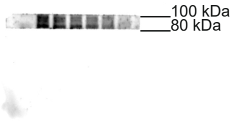

HCC1937
GAPDH

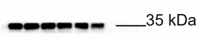

Data S7

Hs758T
FN1

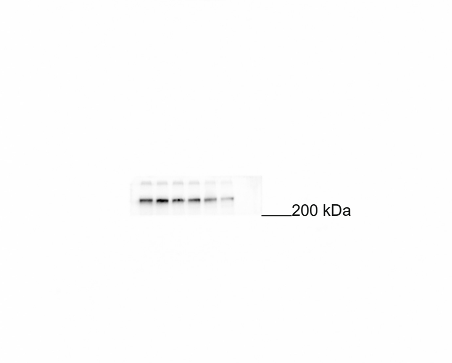

Hs758T
VIM

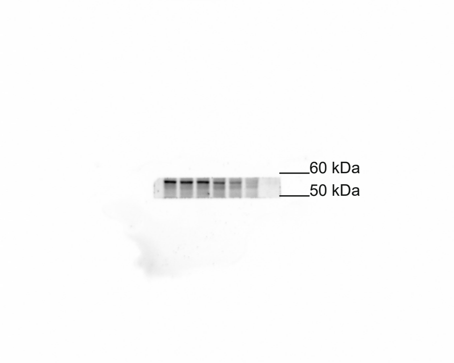

Hs758T
GAPDH

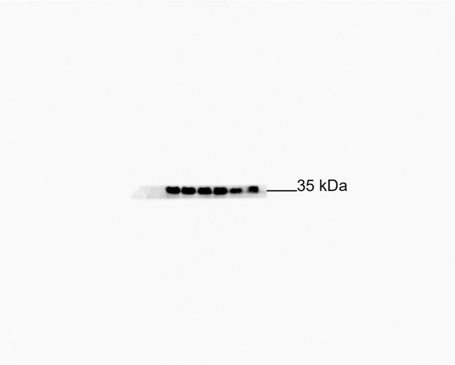

HCC1937
FN1

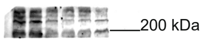

HCC1937
VIM

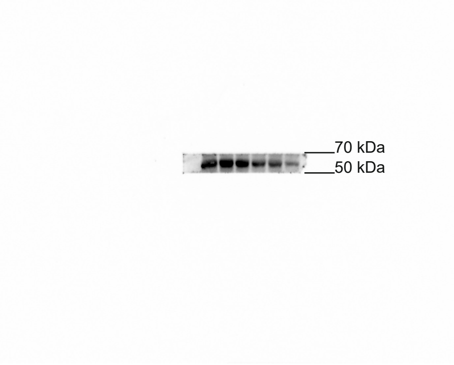

HCC1937
GAPDH

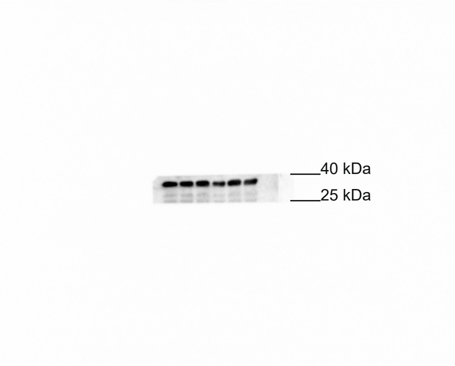

Supplement: Document S1. Figures S1–S6 and Data S1–S7 [file mmc1.pdf]
